# Black Clinicians’ Perceptions of the Cultural Relevance of Parent–Child Interaction Therapy for Black Families

**DOI:** 10.3390/ijerph21101327

**Published:** 2024-10-08

**Authors:** Erica E. Coates, Sierra Coffey, Kaela Farrise Beauvoir, Emily Aron, Katherine R. Hayes, Felipa T. Chavez

**Affiliations:** 1 Department of Psychiatry, Georgetown University Medical Center, Washington, DC 20007, USA; emily.j.aron@gunet.georgetown.edu (E.A.); katherine.hayes@georgetown.edu (K.R.H.); 2 Department of Psychology, Georgetown University, Washington, DC 20057, USA; sac326@georgetown.edu; 3 Department of Counseling, Clinical and School Psychology, University of California-Santa Barbara, Santa Barbara, CA 93106, USA; kaela@ucsb.edu; 4 School of Psychology, Florida Institute of Technology, Melbourne, FL 32901, USA; chavezf@fit.edu

**Keywords:** parent–child interaction therapy, black families, cultural adaptation

## Abstract

Parent–child interaction therapy (PCIT) is a highly efficacious, evidence-based treatment for children with disruptive behaviors and their families. PCIT is a dyadic therapy designed to improve parent–child relationships and decrease children’s behavioral problems. PCIT research specific to Black families is currently sparse. Given findings that Black families have a higher attrition rate and demonstrate fewer significant improvements in parental well-being outcomes, we sought to assess clinicians’ perceived cultural alignment of PCIT with Black families. We conducted individual interviews via Zoom with 10 Black clinicians, trained in PCIT, who had experience treating Black families using PCIT. The research team generated the following themes using thematic analysis: cultural misalignment, manualization, barriers to treatment, generational patterns of discipline, racial considerations, and protocol changes. Findings indicate that Black clinicians have identified various points of cultural misalignment in providing PCIT with Black families, for which they have modified treatment or suggested changes to improve cultural sensitivity. Collating suggested clinician modifications to inform a cultural adaptation of PCIT for Black families may contribute to a reduction in the attrition rate and improvement in outcomes for Black families participating in PCIT.

## 1. Introduction

Parent–child interaction therapy (PCIT) is a behavioral parent training (BPT) program that is considered to be evidence-based in effectively treating young children with disruptive behaviors via a family coaching model [1]. PCIT is distinct from other BPTs in that PCIT utilizes real-time, in vivo feedback to coach parents through specific interactions with their children to promote a secure parent–child relationship and decrease children’s disruptive behaviors [2]. PCIT has been found to improve parenting skills, decrease child behavior problems, and generalize benefits to untreated siblings [2]. While PCIT has shown efficacy with certain populations, there has been some debate as to whether the evidence exists to consider PCIT evidence-based for Black families [3]. Since the development of PCIT with White families in a rural context, an overwhelming majority of PCIT studies in the U.S. have been conducted with only-White or majority-White samples [3]. Further, there is a particular paucity of research in determining PCIT’s efficacy with Black families and whether adaptation is needed [4]. Of the PCIT studies conducted with at least some Black participants, research has shown a number of disparate outcomes between Black families and families of other racial/ethnic backgrounds. These findings include higher levels of attrition for Black families, longer treatment durations needed to achieve proficiency in the PCIT skills when compared to typically understood treatment lengths of 12–16 weeks [5], and no mitigating impact on parental stress or depression, which was observed in other families [6,7,8]. We sought to address whether a cultural adaptation of PCIT is warranted and assess the specific areas of adaptation needed.

### 1.1. Parent–Child Interaction Therapy

PCIT is a heavily manualized BPT intervention [9]. Before beginning PCIT treatment, clinicians lead caregivers through a pretreatment assessment to determine appropriateness of fit, including a clinical interview, observation of caregiver–child play, and the caregiver’s completion of questionnaires like the Eyberg Child Behavior Inventory [10]. Clinicians document real-time observational data via the Dyadic Parent–Child Interaction Coding System (DPICS), a standardized behavioral observation system developed to assess the quality of caregiver–child interactions [11]. Clinicians’ consistent monitoring of caregivers’ behaviors during sessions via the DPICS metrics helps to track progress in skill acquisition over time [11].

The PCIT treatment protocol that clinicians follow outlines the assessment and treatment sessions. The standard PCIT protocol consists of two treatment phases: child-directed interaction (CDI) and parent-directed interaction (PDI). CDI focuses on building a secure parent–child relationship by instructing and coaching parents to allow the child to lead the play. During CDI, the parent is coached to use selective attention to positive behavior and withhold attention for minor negative behavior while avoiding commands, questions, and criticisms. PRIDE skills, an acronym for a set of behavior skills that caregivers are taught and coached on during sessions, emphasize caregivers doing five things: giving children specific praise (P), reflecting what the child says (R), imitating the child’s actions and mannerisms (I), describing what the child is doing (D), and showing enjoyment in play with the child (E).

PDI focuses on teaching parents to provide structured and consistent discipline strategies to their children, including a specific time-out procedure. The time-out procedure involves a time-out chair sequence as well as a time-out room sequence that is used if the child does not remain in the time-out chair for the designated period. In order to further standardize treatment, the PCIT manual includes scripts on how to teach the PCIT concepts to caregivers. Further, there is an emphasis on changing verbal and physical interactions between caregivers and their children via live coaching and daily homework assignments. While treatment fidelity is key for proper PCIT delivery, researchers and clinicians have developed specific adaptations over the past two decades to account for cultural nuance needed to make PCIT effective with specific populations that were not captured in the original protocol.

### 1.2. PCIT Cultural Adaptations

PCIT researchers have made notable advances in adapting PCIT to increase the acceptability and effectiveness of PCIT with various populations and cultures (e.g., Mexican Americans and Native Americans) within the U.S. [4]. However, these research efforts into the effectiveness of PCIT remain at varying stages, wherein research with some populations remains in the recommendation and pilot stages [12,13] and other research investigations have produced systematically developed adaptations and examined the efficacy of these adapted interventions in randomized controlled trials [14]. For instance, McCabe et al. developed and validated Guiando A Niños Activos (GANA), a PCIT adaptation intended to meet the cultural nuances of Mexican American families [14,15,16].

The cultural adaptation process to develop GANA incorporated a structured procedure including reviewing literature, gathering input from Mexican American families and clinicians who serve the intended population, and incorporating feedback from stakeholders after a model was proposed [15]. In a randomized controlled trial (RCT), GANA was compared against treatment as usual (TAU) and the standard PCIT protocol [14]. Results from the RCT showed that both GANA and PCIT were more effective than TAU at improving children’s externalizing behavior problems and parenting stress, as well as having higher observed labeled praises and parental satisfaction rates. However, only GANA was also significantly more effective at improving all other parent-reported outcome measures over TAU and was shown to elicit significantly more father participation than TAU [14]. In a one-year follow-up study, GANA, but not PCIT, was found to significantly improve 6 out of 10 outcomes measured when compared to TAU, including externalizing and internalizing behavior [16]. Although there were no significant differences between GANA and PCIT on most studied outcomes, GANA significantly outperformed PCIT in improving children’s internalizing symptoms at follow-up. These findings indicate that a successful cultural adaptation likely requires attention to language as an identity marker, barriers to treatment and completion, and a group’s historical relationship to mental health services.

Researchers have also begun the cultural adaptation process with other minoritized populations in the US, including Native American families [12] and Puerto Rican families [13]. Though cultural adaptations of PCIT are ongoing for multiple minoritized populations in the U.S., scant research has focused on the need for a cultural adaptation of PCIT for Black families [4]. More recently, there has been evidence of promising treatment outcomes for a more personalized tailoring approach to PCIT, such as MY PCIT [17]. The MY PCIT adaptation focuses on the use and important need to incorporate preliminary assessments with families during the intake process focused on factors that may influence engagement and completion rates for ethnically minoritized families. The family’s assessment findings are used to specifically tailor PCIT treatment to meet the cultural needs and values of the family while maintaining fidelity to the underlying treatment model. Inherently, the concept is a good one that transcends various cultural affiliations. One concern with this approach is its overly general nature of the cultural assessment that may fail to capture the unique issues relevant to Black families, such as justified mistrust in research or healthcare settings, structural language considerations, or differing ethnic-racial socialization practices. Specifically, ethnic-racial socialization is a critical element in parenting in the formation of healthy racial identity for Black children [18], for which the literature speaks to positive child outcomes behaviorally, cognitively, academically, and socially [19,20]. Further, while MY PCIT identifies various assessment factors relevant to ethnically minoritized families as outlined in the research literature, it does not account for the dearth of research on barriers and facilitators to engagement specifically for Black families with young children.

### 1.3. PCIT with Black Families

Eyberg (2005) concluded that PCIT was an evidence-based treatment for young, white children with behavior disorders, but that there was a lack of evidence for PCIT being efficacious for Black families of young children with similar behavioral challenges. Nearly 20 years later, only a handful of peer-reviewed articles have been published examining outcomes for PCIT in majority-Black samples [7,21,22,23]. Moreover, only one article, a retrospective archival study of 18 Black parent–child dyads, has been published with a 100% Black sample [7]. Although researchers found that most Black mothers who met completion criteria for PCIT reported a significant reduction in their children’s behavioral problems, there was not a significant reduction in either maternal stress or depression from pre- to post-treatment [7]. Moreover, Black families demonstrated an overall attrition rate of 56%, with 70% of non-completers dropping out following the assessment and before beginning treatment [7]. The attrition rate found for Black families participating in PCIT was considerably higher than the 36% attrition rate found in a previous study with a majority-White sample [24].

In another study, researchers conducted a community-based pilot of PCIT with a focus on eliminating barriers to treatment for families with low-income and ethnically minoritized children (*n* = 14, 50% non-Hispanic Black children; 21% Black Hispanic children) [23]. Specifically, they sought to eliminate barriers to treatment by providing free transit cards for public transportation to sessions, offering to have sessions at a local daycare instead of at community mental health centers, accommodating participants’ requests for evening sessions, and exercising a liberal absence policy. Despite these efforts, overall attrition in the study was 71%. Researchers found that there was a clinically significant change in behavior among the children that completed treatment. However, researchers postulated that factors related to low SES, self-referral status, perceptions of culturally misaligned treatment, and high levels of treatment barriers affected attrition.

A couple of studies have compared PCIT to other early childhood-focused evidence-based treatments (EBTs) with majority-Black samples. For example, researchers compared PCIT and the Chicago Parenting Program in a majority-Black sample (*n* = 80 in the PCIT intervention; 72.5% Black caregivers) [22]. Clinicians rated parents participating in PCIT as more engaged than parents participating in the Chicago Parenting Program, with treatment completers attending 31 sessions on average. However, the overall attrition rate was 69.4% for families who attended at least one PCIT session. Identified barriers for families included lack of transportation and childcare for non-participating siblings; both were provided by the clinic for families in need. Lastly, a recently published study focusing on EBTs delivered to families in homeless shelters compared a time-limited version of PCIT (*n* = 70 in the 12-session PCIT intervention, 77% Black children) to time-limited Child–Parent Psychotherapy [21]. Overall, researchers found both interventions to significantly reduce parenting stress and increase positive parental verbalizations. However, only time-limited PCIT was clinically significant in reducing children’s externalizing behavior problems and parents’ negative verbalizations among treatment completers, although attrition from the point of intake was 56% (52% for those who began treatment). Moreover, researchers found that mothers who participated in time-limited PCIT had significant reductions in stress and negative verbalizations as well as significant increases in positive verbalizations compared to mothers in time-limited Child–Parent Psychotherapy. While a few studies have been conducted with majority Black samples, the focus has not been on cultural or racial differences in outcomes, and there is still much research needed to determine whether a cultural adaptation of PCIT is warranted for Black families.

### 1.4. Current Study

Since Eyberg’s (2005) call to action, few studies have taken up the mantle of examining the efficacy of PCIT in treating mental health and behavior outcomes in Black families. While Black families have been included in some PCIT research studies, there is limited research focusing specifically on the experience of Black families completing PCIT or the effectiveness of PCIT within this population. As Black people are at risk for overdiagnosis of disruptive behavior disorders [25,26] and have high PCIT attrition rates as well as disparate outcomes [7,22,23], there is a clear need to explore the perceived suitability of PCIT for Black families. Asking clinicians about their implementation experiences is one way of understanding how EBTs like PCIT are perceived and adapted to fit diverse communities [27]. Our study is informed by a cultural adaptation process that included collecting data from clinicians who worked with the intended population to assess cultural congruence and accessibility of the intervention [14]. In the current study, researchers conducted qualitative interviews with Black-identifying clinicians trained in PCIT about their experiences providing PCIT to Black families in order to contribute meaningful insights from clinicians in the field serving the Black community. The goal of the study was to assess Black clinicians’ perceptions of the cultural fit of PCIT for Black families and modifications needed to improve the cultural alignment. With this study, researchers hoped to explore two primary lines of inquiry:

- How do Black clinicians perceive the cultural alignment of PCIT with Black families, and what are the barriers to treatment accessibility and completion?
- What modifications do Black PCIT clinicians suggest are needed to increase the cultural alignment of PCIT with Black families?

## 2. Materials and Method

### 2.1. Participants

Participants were recruited from the PCIT international listserv, and one participant was referred to the study group by another clinician. Eligibility criteria for the study included being trained in PCIT, self-identifying as Black or African American, and having provided PCIT to at least one Black family. Participants were Black clinicians (*n* = 10) who provided PCIT to between 2 and 40 Black families (*M* = 15.3, *SD* = 14.7). All participating clinicians identified as female and ranged in age from 30 to 62 years old (*M* = 38.2, *SD* = 9.4). Half of the participants had doctorate degrees, and the other half had master’s degrees. All were licensed clinicians, with 40% of participants being licensed psychologists, 40% licensed in social work, and 20% licensed in counseling. Participants had between 5 and 40 years of experience providing therapy (*M* = 12.3 years, *SD =* 10.2) and between 2 and 11 years of experience providing PCIT (*M =* 5.4 years, *SD* = 3.6). Participants’ estimated percentage of Black families who successfully completed PCIT treatment with them ranged from 0% to 100% (*M =* 51.0 percent, *SD* = 34.1). See Table 1 for participant characteristics using pseudonyms.

### 2.2. Interview Questions

The development of interview questions for this study involved an iterative process including members of the research team and a consultant with expertise in culturally adapting EBTs for Black families. Beginning with a literature review on previous PCIT cultural adaptations and treatment barriers for Black families conducted by the research team, the first author drafted an initial set of questions according to the specific stages of PCIT. The first author circulated the drafted questions to research team members and the consultant to elicit feedback. The first author convened multiple meetings with the research team to collaboratively refine and finalize the questions. During these discussions, the team sought to ensure that all questions were clear, relevant, and aligned with the research objectives. The research team revised questions as needed and agreed on the final set of questions (see Table 2 for a list of interview questions).

### 2.3. Procedure

Clinicians were recruited through a flier that was disseminated through the PCIT International listserv in January 2023. The clinicians were screened via email to confirm eligibility criteria. Clinicians that were screened eligible were scheduled for a virtual interview time with a member of the research team. PCIT clinician interviews were conducted in February and March of 2023. Clinicians completed informed consent via Docusign prior to the interview. To join the interview, participants logged onto the Zoom link they received after selecting an interview time. The interviews lasted approximately 48 min, on average. We compensated clinicians with a 100 USD e-tango gift card for their time following completion of the interview.

### 2.4. Researcher Positionality

The authorship team is composed of undergraduate, graduate, and early and mid-career scholars. Two scholars self-identify as Black or African American, two as White, one as Afro-Latina, and one as Biracial (Black and White). All members of the research team are cisgender, heterosexual women. Interviews were conducted by four self-identifying Black, female, undergraduate, and graduate student members of the research team. All interviewers had direct experiences with, knowledge of, and shared interests in Black families’ cultural strengths and wellbeing. All coders had overlapping identities as Black women with study participants, and two of the three coders were also PCIT clinicians. Study recruitment, screening, interviews, and data cleaning were conducted under the guidance of the first author, a Biracial, cisgender, female clinical psychologist and director of a research collaborative focused on culturally sensitive mental health care of Black people and families. Data coding and analysis was conducted under the guidance of the senior researcher, an Afro-Latina, cisgender, female clinical psychologist and director of programs focused on disseminating PCIT and Teacher Child Interaction Therapy to underserved communities.

### 2.5. Trustworthiness

Trustworthiness in this study was established in several ways. Regarding the data collection process, credibility was ensured by the inclusion of all faculty researchers on the membership-only PCIT International listserv, through which participants were recruited. The research team established and followed a standardized protocol for data collection, and interviewers were trained in the facilitation of structured interviews. The senior researcher was versed in qualitative analyses, developed the codebook, and trained and guided the research team in all aspects of the data analysis. Furthermore, the Black, female interviewers evidenced interest and familiarity with the values and norms within the Black community, which helped to ensure the comprehensiveness and richness of the data collected by demonstrating an understanding of participants’ responses.

### 2.6. Data Analytic Plan

Given that existing research on this topic is scant, researchers drew from multiple methodologies to forge a comprehensive data analytic plan. Recognizing that Black points of view are lacking in PCIT research specifically and intervention research more broadly, a social constructivist theoretical approach was used wherein participants’ experiences and perceptions are valued and prioritized as important and critical data [28]. As described below, an inductive, codebook-oriented thematic analysis approach was taken to derive a preliminary codebook from a subset of participant interviews [29]. More specifically, a bottom-up strategy [30] was used in which a codebook was generated based on common holistic themes emerging across the interview dataset, for which codes were generated.

Clinician interviews were recorded and transcribed via Zoom and stored in Box. Before beginning analysis, members of the research team reviewed each video and cleaned each transcript for accuracy. After all the transcripts were cleaned, two black, female members of the research team independently reviewed the transcripts and compiled notes, which the senior researcher used to create the initial codebook. The senior researcher met with the coders via Zoom over several sessions to refine the codebook through the generation of new codes and synthesis of similar codes. While the original codebook was created using a bottom-up qualitative data-driven process, codebook refinement utilized a top-down process informed by the senior researcher’s wealth of expertise with the PCIT treatment modality, servicing Black families in treatment for close to 20 years, and the research literature associated with both. Thus, this top-down process was consistent with a deductive reflexive analytic approach for interpreting responses in the refining of the codebook [31,32]. See Appendix A for a copy of the final codebook [33].

The initial data analytic plan was to use a codebook approach to thematic analysis to establish a set codebook and achieve interrater reliability (IRR) in order to split the interview coding among the team members. However, IRR was not achieved after multiple rounds of codebook review given the wide array of participant experiences, which was more than initially anticipated. Therefore, researchers shifted to a consensus coding approach wherein codes were reviewed and approved by at least 3 coders. If segments of an interview did not fit into pre-existing codes, new codes were created and incorporated into the analysis as the coders read and reread each interview. Subsequently, interviews were recoded using the final version of the codebook to ensure all potential codes were assigned to each relevant segment.

After coding was finalized, total count scores were calculated for how many clinicians mentioned each code across interviews. Further, totals were calculated for the number of times each Black clinician mentioned a single code. Themes were selected based on those receiving the highest count scores. That is, themes that appeared most frequently across transcripts were considered the most prominent themes for subsequent analysis and reporting. Lastly, quotes that especially captured the essence of the themes, subthemes, and codes were identified for inclusion in the narrative.

We sought to achieve further transactional validity of our findings through a process of member-checking [34,35]. Researchers disseminated preliminary results to study participants to confirm the accuracy of themes generated from their interviews and to provide an opportunity for feedback. We sent participants a Powerpoint slide deck outlining an overview of the project, the themes and subthemes we generated based on the data, and a summary of the conclusions drawn from the data. We emailed participants with reminders multiple times over a 5-week period to encourage their response. Three of the 10 Black clinicians responded with positive feedback about the results, and there were no suggested edits.

## 3. Results

Results of our thematic analysis of the clinician interviews are illustrated by the following six themes generated by the researchers: cultural misalignment (*n* = 10), manualization (*n* = 10), barriers to treatment (*n* = 10), generational patterns of discipline (*n* = 9), racial considerations (*n* = 10), and protocol changes (*n* = 10). The majority of the themes (i.e., cultural misalignment, manualization, barriers to treatment, and generational patterns of discipline themes) were derived directly from the codebook, as they were mentioned by the highest number of clinicians, as were the subthemes described within the themes. For two themes (i.e., “racial considerations” and “protocol changes”), we subsumed two themes with overlapping or complementary content under an umbrella theme and included the highest-rated subthemes across the themes in our results. Specifically, “racial considerations” is the summation of “bilingualism/biculturalism” and “systemic racism, discrimination & PCIT”, and “protocol changes” is the summation of “suggestions” and “clinician attributes”. The research team determined that combining themes in these two cases best showcased themes and subthemes with high count scores in a parsimonious fashion. Each theme and subtheme are further described below using pseudonyms for participants’ names. See Table 3 for a summary of themes and subthemes.

### 3.1. Cultural Misalignment

#### 3.1.1. Cultural Sensitivity

A majority of the clinicians (*n* = 9) stated there is a need for cultural sensitivity in PCIT in relation to working specifically with Black families. Increased cultural sensitivity encompasses a number of different areas described by clinicians, including helping alleviate barriers Black families face in completing treatment, tailoring the language to be more accessible to Black families, and recognizing that the sociocultural context that Black families experience is vital information that is necessary for a thorough and effective treatment plan.

All of the clinicians (*n* = 10) discussed how PCIT lacks sufficient attention to helping Black families manage certain systemic and structural barriers that they are more likely to face than their White counterparts, including limited access to community and financial resources, as well as increased caregiver stress. Aisha, a 32-year-old clinician with 10 years of PCIT experience, shared that Black families “…just have either a lot of responsibilities or difficulty, like you know, with child care for the other child, those types of things”. Sade, a 38-year-old clinician with 4 years of PCIT experience, also expressed that PCIT should acknowledge that “some Black families are… up against a few more obstacles than other [racial] counterpart families, and I think to be sensitive to that”.

All of the clinicians (*n* = 10) described the need for more cultural sensitivity to be applied to the language used in PCIT, and more than half of the clinicians (*n* = 6) expressed the need for the DPICS coding to be updated to include modifications that allow for more cultural flexibility. Monica, a 30-year-old clinician with 7 years of PCIT experience, commented that she thinks that “[coding] can hinder and sometimes extend the CDI phase where we’re not meeting mastery criteria because of these little things that are not little things”. Clinicians commented that language is too heavily weighted for determining when caregivers meet target criteria and that other nurturing behaviors also demonstrated by caregivers should be considered as well, particularly regarding ways that caregivers connect positively with their children.

Almost all clinicians (*n* = 9) felt a need for more explicit cultural modifications in the manual and in the protocol. Clinicians described that certain aspects of PCIT are counter to Black parenting style, which may be perceived as more authoritarian. The contrast between parenting approaches can lead to resistance from Black families. For example, Jamila, a 38-year-old clinician with 3 1/2 years of PCIT experience, described the following situation she has observed working with Black families:

We have families who are very eager and wanting to learn and do things differently, but sometimes what we are encouraging… PCIT goes against the culture if that makes sense. And so there is pushback and misunderstanding about utilizing these principles and how it can be effective for their families.

In addition to outlining several areas for needed increased cultural sensitivity, a majority of the clinicians (*n* = 7) acknowledged that certain clinicians of color within the PCIT community have been advocating for the increase in PCIT’s cultural sensitivity over the last few years. When discussing how the term “mastery” was not culturally sensitive, Monica shared the following, which acknowledged that there is change happening linguistically and structurally within PCIT: “There was this whole conversation on how… PCIT International had to stop using ‘master’ trainers because that was problematic. Yeah, because they were all White”. Monica was potentially alluding to the PCIT International town hall following the murder of George Floyd where PCIT clinicians of color raised that the word “mastery” has connotations of racial subjugation that date back to slavery and may be perceived by Black clients as harmful. As a function of that town hall where these issues of cultural insensitivity to terminology were raised, PCIT International replaced “mastery” criteria with “target” or “skill” criteria’ and revised the title of “master” trainers to “global” trainers in 2020. Although PCIT International updated the language used in the treatment protocol manual from “mastery”, this change has not been universally adopted by all PCIT therapists given its inconsistent dissemination.

#### 3.1.2. Child-Directed Interaction

Clinicians (*n* = 7) expressed their perceptions that certain aspects of the CDI phase of treatment were not culturally aligned with Black families. Specifically, some families grappled with the idea of allowing their child to lead, and clinicians commented on experiences where families felt like playtime was not a necessary component of treatment. Monica shared her idea of what a Black family’s perspective may be regarding CDI:

This play feels like a luxury and not a necessity. Where [caregivers say] ‘You need to be learning, you need to be practicing. You need to be doing these things’. And therapy also kind of feels like work, and so trying to shift that mindset [into] ‘So this is playtime, and that it’s necessary’.

Monica stated that the Black families she works with tend to focus on teaching as opposed to playing during CDI, and that Black families tend to emphasize educating their children more than playing with them in her experience. The focus on allowing children to lead the play during special times may counter Black parents’ typical leanings.

Clinicians (*n* = 6) also identified that several of the Black families they have worked with struggle with the ignoring skills. Sade, a 38-year-old clinician with 4 years of PCIT, mentioned that one of the Black families she worked with stated that by ignoring the child, it was “reinforc[ing] that the kid is getting away with it, and that this is okay”. Sade further explained that the client doesn’t “want them [the child] to think that yelling or something like that is okay”. Nia, a clinician with 14 years of PCIT experience, described how she perceives the ignoring skill to be culturally misaligned with Black families:

It’s around frustration… of like ignoring behaviors. A lot of times in Black families, we believe that it’s disrespectful when a child is acting in a certain way or behaving in a certain way, and therefore we need to address it directly. And so the idea of ignoring a behavior for such a young child culturally doesn’t always feel like it fits.

Half of the clinicians (*n* = 5) also reported Black families struggling with the idea of providing frequent labeled praises to their children. For example, Monica noted a caregiver feeling like they are being fake or disingenuous when praising her children:

One of the things that she [the caregiver] had mentioned was about the praises and feeling like she was being fake or that she wasn’t being genuine by just praising her kid all the time, not for no reason, but that they just didn’t feel it was natural.

Another clinician noted additional PRIDE skills that could be encouraged outside of labeled praises. Specifically, Kamilah, a 62-year-old clinician with 11 years of PCIT experience, suggested that physical movement could have cultural relevance for Black families:

Everything is not about a labeled praise; you know they have to find other ways to add Black culture to like, even if it’s a dancing portion or do something. That’s what our kids like to do. They like to move. They like to dance. Yes, of course they like to play, but it’s just sitting there playing.

Half of the clinicians (*n* = 5) mentioned that Black caregivers may perceive their parenting style being threatened during CDI and that CDI principles may invalidate their sense of self as a parent and potentially their cultural norms. For example, Jamila described CDI as “really challenging [Black caregivers]” by “changing some of their ways of thinking, changing some of the ways they’re interacting” with their children. There was a sense from some clinicians that expecting caregivers to change their parenting styles too drastically may ultimately lead to attrition. Joan, a 34-year-old clinician with 10 years of experience, described this sentiment below:

A lot of times we ask families to change their parenting style. Like to think about how you approach, how you interact with your child, and how you parent your child differently, and that is a big ask. And sometimes… I’ll say that in CDI that is a big ask that I’m asking you to rethink how everyone around you thinks about parenting and I think that is… where I lose them is that it’s too big of an ask.

Overall, clinicians shared their perspectives on how several aspects of CDI, including the PRIDE skills, ignoring, and special time focused on following the child’s lead during play, may counter Black families’ cultural norms and contribute to attrition. Clinicians described strategies such as coaching and modeling through a parallel process, providing space for communicating about parents’ perceptions, and adding skills that may be better culturally aligned to increase Black parents’ acceptability of this phase of treatment.

#### 3.1.3. Parent-Directed Interaction

Just over half of the clinicians (*n* = 6) discussed the misalignment between the PDI Phase and Black families. Clinicians described that PDI and the focus on time-out are not what Black families are expecting in treatment. Sade, a 38-year-old clinician with 4 years of PCIT, stated that the mismatch in expectations can lead to attrition for some of her Black families: “So I think that we definitely still have opportunities to lose families [during PDI], and some of them do [drop out]. Yeah, because they feel like, again, maybe this– it wasn’t what they were expecting”. Nia, a clinician with 14 years of PCIT experience, also described Black families dropping out during PDI because they don’t have an accurate assessment of the level of stress that time-outs can cause. Nia further elaborated on Black families dropping out when clinicians lack transparency “Because it’s not what people expect. If [clinicians] just go ‘Oh a little time-out to sit and think’ you know it, it’s not always that”.

Other clinicians discussed the skepticism that Black parents may experience during PDI. Zara, a 36-year-old clinician with 2 years of PCIT experience, described how she often needs to “prove” to her Black families that PDI techniques, such as effective commands and time-out, actually do work:

So sometimes in the PDI Teach… I notice that I have to do some convincing. So I have to show research and data. I have to prove that somehow, like what I’m saying, has merit and that they can really see a difference when they use a different strategy during the Teach phase session.

When asked about why people drop out of the PDI Phase, clinicians often cited that PDI takes too long. Kamilah, a 62-year-old clinician with 11 years of PCIT experience, added that “Maybe it [PDI] was too long for them, or they were just tired of the situation altogether”. One of Kamilah’s clients shared with her that because PDI is so long, it’s not culturally appropriate:

I got the one parent that just finally, she wasn’t giving it everything, and then she was like, “You know this is not culturally sensitive. We were already on PDI 7. They don’t even have a spot for it on the paper for that”, so it was going to be very difficult…so I just stopped it all together.

Because of the length of PDI, some clinicians (*n* = 4) stated that families may not see the need to reach goal criteria for graduation before terminating treatment. As articulated by Nia, a clinician with 14 years of PCIT experience, “Some [Black caregivers] will feel like they have the basic skills, so they’re going to, like, continue it on their own. And so they stop coming”. Clinicians described that after learning the PRIDE skills and forming a better relationship with their child during CDI, some Black families perceive PDI as unnecessary, particularly in the context of misaligned techniques. To counter this, some clinicians described using research to convince Black families of the effectiveness of this stage of treatment, and other clinicians suggested that there be more preparation built into PCIT before PDI begins.

#### 3.1.4. Time-Out

Almost all clinicians (*n* = 8) mentioned that Black families had concerns regarding the time-out procedure. Aisha, a 32-year-old clinician with 10 years of PCIT experience, noticed that one of the families she worked with “Had this idea like time-out [was] for White families… Like ‘we don’t do that like [child] is, you know, being disrespectful”. Clinicians (*n* = 4) reported that according to Black families, *time-out* may not be an effective discipline strategy for parenting their young Black children, for whom high compliance is not only a cultural demand but could be a potentially life-saving necessity when dealing with systemic authority figures, consistent with Black parental ethno-theories of ethnic-racial socialization. Accordingly, nearly half of the clinicians (*n* = 4) commented that Black caregivers have low tolerance for disrespect from their children, hence why caregivers tend to favor discipline strategies that elicit more immediate changes in behavior. For instance, Nia made the following comment regarding caregivers’ concerns about time-out as a realistic strategy for their Black children:

And I think that one of the things that sometimes comes up is people’s belief that ‘Well no one else is gonna just give my child a time-out’. Like the world is hard, right? Police aren’t gonna you know, know all these things that they need to understand and know how to help themselves and regulate themselves even when someone [e.g., the child] is hitting them, even when someone is being really harsh on them. Because the world is harsh on Black children.

On the other hand, some clinicians (*n* = 3) had experience with clients who perceived time-out, particularly the time-out room, as “abusive” or “triggering”. Black clinicians described some of their Black clients as equating the time-out room with abuse because confining their child in a room felt uncomfortable. Similarly, clinicians expressed concern regarding their Black families’ willingness to implement the time-out procedure. Monica reflected on her initial reactions to learning about PDI when she was training to become a PCIT therapist:

No one’s gonna do that. I can just imagine [Black] families like being worried about their neighbor calling CPS [child protective services] on them because this kid is screaming for 20 min straight being locked in a room. So, some of those things felt a little like uncomfy.

Additionally, clinicians (*n* = 2) expressed concerns regarding Black families having the appropriate infrastructure for the proper implementation of time-out procedures within their home. In many households, especially those with multiple children or multiple families, there are limited private spaces to practice the PCIT time-out procedure, which requires a separate space for the time-out room. In Sade’s experience, many Black families “don’t have the same setup at home… if there’s a time-out chair in a time-out room, [Black parents] don’t have that set up at home so [they’re] kind of [like] ‘What should I do?’” Clinicians (*n* = 6) discussed allowing the space in sessions for families to share their concerns related to the time-out procedure.

#### 3.1.5. External Judgment

Clinicians (*n* = 6) elaborated on the influence external judgment from family, friends, and the Black community had on Black clients completing treatment. Such judgments about the ways caregivers are choosing to parent their children can be a stressor for Black families participating in PCIT. Monica described the influence external family members have on Black clients:

A lot of times, we’re fine in session. But then it’s, ‘Well grandma’s gonna come over, and she’s gonna have a problem with this and that’ or ‘[I] can’t explain to my sister that we’re not whooping him—we’re doing this instead’.

A lot of clinicians talked about the struggle their Black clients face in wanting to try different parenting techniques but also having to deal with the criticism of others close to them who are unfamiliar and unsupportive of the PCIT techniques.

In addition to having to explain PCIT techniques to family and friends, clinicians noted caregivers’ trepidation about using PCIT within the larger Black community. For example, a client of Joan’s discussed her concerns with using PCIT skills in majority Black spaces: “Do you know how I’m going to look if I go to church, and I’m telling my son to sit on time-out?” Because one’s sense of community can be an incredible resource for Black families, judgment from one’s support system can be a large barrier for the successful completion of PCIT.

Black clinicians (*n* = 8) discussed empowering their families, providing space for Black caregivers to process their experiences using PCIT strategies within their communities, role-playing, and providing resources to caregivers that they could share in discussions with those in their network regarding why they are using specific parenting practices. Jamila discussed empowering her families to be confident in their parenting decisions while appreciating how important community is for Black families:

We know how big the family structure is in our community, and you know, being able to place value on that, but also give them [caregivers] that, you know, independence to be able to stand up to some of the things that may prevent them from progressing.

In the PDI phase in particular, Jamila thinks it is important to give families “words and motivation to be able to deal with outside influences”. Clinicians discussed empowering Black families by emphasizing that they are doing the best they can in seeking treatment to better manage their children’s behaviors, and they have their children’s best interests at heart.

### 3.2. Manualization

#### 3.2.1. PCIT Rigidity

All clinicians (*n* = 10) commented on how the rigidity of PCIT lacks cultural sensitivity with Black families. Clinicians specified various instances of PCIT rigidity, including in the manual (*n* = 5), skill criteria (*n* = 5), and assignments (*n* = 3), in addition to the two other subthemes: language (*n* = 7) and DPICS coding (*n* = 4). Given the perceived rigidity of the intervention, multiple clinicians (*n* = 8) discussed tailoring the protocol to increase the likelihood of families’ successful completion. Latoya described her experience tailoring PCIT when working with Black families:

It did feel PCIT, at times, in general does feel cookie cutter at times as well, so I did find myself having to kind of tailor it to kind of fit my style and just fit the style of the [Black] families that I work with.

Other clinicians (*n* = 4) also described being selective regarding which families they would offer PCIT given its perceived rigidity. Kamilah stated that because of the intensive requirements of PCIT, such as daily homework, weekly parent–child sessions, and meeting skill criteria goals, she is mindful about which families she offers PCIT:

I have to pick and choose who I think they can be successful with [it] because I have some parents that truly need PCIT. But because, you know, they’re so resistant that I know that they are not gonna go through that whole rigid requirements and having to say what you know was required to be said and doing the homework and all that.

Overall, clinicians described strict PCIT requirements as an impediment to Black families’ successful completion.

#### 3.2.2. Language Concerns

In particular, most clinicians (*n* = 7) commented on cultural issues with the language used in PCIT. Clinicians shared that they and their clients believe that some words and phrases used in PCIT are “dated”, “awkward”, or “problematic” (n = 4), which makes it difficult to understand treatment. Clinicians explained that because PCIT is especially language-based, Black parents feel the need to use the PCIT-suggested language despite typically communicating with their children very differently. Latoya described the sense of anxiety that language brings to her clients, “The disconnect between once again the language and the need to sound perfect I think wasn’t helpful”. The focus on language is significant in PCIT as the ways in which parents speak to their children during sessions determine whether parents advance to the next stage of treatment, which is further discussed below.

More than half of the clinicians (*n* = 7) mentioned Black families having trouble implementing PRIDE skills such as behavior descriptions and reflections because it does not resemble how parents talk to their children at home. Kamilah provided her observations of the ways it was harder for Black families to adapt to the language, which would prolong the time for them to reach CDI skills criteria:

They were struggling with trying to make mastery because, and I think again, it may have been about the language because they wanted to be… it is very specific. You have to have label praises and the behavior descriptions and the reflections.

Clinicians (*n* = 8) discussed tailoring treatment by describing concepts in a different way to increase Black families’ understanding. For example, Ayanna, a 35-year-old clinician with 3 years of PCIT experience, described the following: “I think what made it more culturally competent was the way I would have to modify, you know, my language and certain things to make it feel more culturally competent”.

#### 3.2.3. DPICS Coding Incompatibility

The majority of the clinicians (*n* = 6) mentioned how DPICS coding lacks cultural sensitivity. Due to a lack of attention to African American Vernacular English (AAVE) in DPICS, Black caregivers’ praises are often coded as commands. Nearly half of the clinicians (*n* = 4) commented about this discrepancy. Three clinicians commented on the phrase “Look at you”, which is often used by the Black community as a term of recognition and praise, technically needing to be coded as a command. Aisha, a 32-year-old clinician with 10 years of PCIT experience, recounted an experience she had multiple times while implementing PCIT with Black families.

I remember another one that gets me every time is like, ‘Look’. Because it’s like it’s a command, technically right, but like my parents, I see it all the time like, ‘Look at you, look at your hair’, and it’s like it’s a compliment.

According to PCIT guidelines in the DPICS research manual, idiomatic neutral phrases can be coded as praise when accompanied by a gesture; however, the guideline does not speak to commands. Thus, this phrase would be coded as a command, especially if it was not accompanied by a positive gesture. All three clinicians felt uncomfortable by this classification, with one clinician even stating that she modified her coding process to count it as praise versus a command.

Monica provided the following suggestion given the noted limitations of DPICS coding with Black families, “I wonder if we should just make a DPICS manual for Black people because there are things that we [Black people] say that our praise may not sound like praise to other people”. Monica expressed that PCIT should incorporate the nuance of how the child reacts to the statement into the coding process to improve its cultural sensitivity with Black families. Overall, clinicians commented that the PCIT protocol, language, and coding lack cultural sensitivity and are treatment-related obstacles preventing Black families from benefiting fully from PCIT because of an inability to move forward in reaching the skills criteria.

### 3.3. Barriers to Treatment

#### 3.3.1. Time Constraints

All clinicians (*n* = 10) mentioned how participating in PCIT requires a significant time commitment for Black families. Going to PCIT treatment sessions often imposes time management difficulties for families, placing them in positions where they have to choose between work, spending time with their other children, and going to therapy. Clinicians expressed concern regarding families’ ability to attend weekly sessions, especially if caregivers were unable to get time off from work (*n* = 7), unable to find childcare for other children (*n* = 5), or reliant on public transportation (*n* = 3). When asked about why Black families often drop out of PCIT, Sade described issues related to the required time commitment:

Going back to like the time commitment, and the barriers that brings about… did I also say, their work schedule of course, pulling kids out of school, and them also having to get off work if their jobs, if their employer supports that, that’s definitely another big thing.

Clinicians noted that time constraints may be further exacerbated among single-parent families and families with multiple children. Because of this, clinicians noted their or their agencies’ attempts to provide families with child-care for other children (*n* = 2) or offering more flexible appointment options (*n* = 4). As described by Zara, “What worked well was that I allowed siblings to come in and I have toys in another room for siblings. That works really well”.

PCIT also requires parents’ time outside of therapy sessions. PCIT participants are highly encouraged to complete daily homework, and most clinicians (*n* = 5) commented that it is difficult for parents to complete daily homework with their other responsibilities. Nia, a clinician with 14 years of PCIT experience, shared that she has never had a Black family complete homework more than 4 days a week. She expressed her understanding regarding the difficulties completing daily homework with the following quote: “This is hard. and it sounds easy. It’s 5 min a day, but it’s a hard thing for [Black families] to implement”.

Lastly, since PCIT is based on meeting skills criteria, it can take families who are not able to attend consistently or complete the homework regularly longer to reach criteria goals. Clinicians (*n* = 5) noted that when therapy becomes prolonged, Black families may become discouraged with the process. For example, Joan recounted her experience with a Black family’s length in treatment: “They’ve been in PCIT for too long, and they’re like, ‘Well, this is taking too long, like what are we doing here’?”.

#### 3.3.2. Socioeconomic Status

Almost all clinicians (*n* = 9) discussed how finances were a hindrance to treatment completion for Black families. The time commitment PCIT requires is challenging for many families’ schedules; however, families with low-incomes especially have a hard time meeting the demands PCIT requires to be effective. When noticing Black families dropping out of treatment, Jamila, a 38-year-old clinician with 3 ½ years of PCIT experience, asked her families their reasons for attrition. She shared the following conclusions: “Poverty and work schedules and things that come along with being [lower] socioeconomic class families, makes it more difficult for [Black families with low-income] to commit to the program and fully graduate”.

Relatedly, multiple clinicians (*n* = 3) noted their clients’ transportation concerns. Not only was transportation costly in terms of finances, but in terms of time as well. Sade shared the following observations from her clinical practice:

Getting to the clinic, transportation is a big issue for some of our families, especially when they’re coming from far away, you know, an hour or more especially, and then maybe even it’s just time of day like it’s–it’s in the daytime when kids are in school. So they got to pull the child out of school to bring them here.

Clinicians also voiced concerns that Black families may not have insurance accepted by the clinic or financial means to pay out of pocket (*n* = 2). Given the structural disparities in the U.S., Black families are overrepresented in lower socioeconomic statuses and in service positions for their jobs that afford less flexibility and benefits, which may limit their ability to fully commit to the completion of PCIT. Inconsistent appointments and homework completion due to competing demands tend to prolong treatment, resulting in family disillusionment with treatment.

### 3.4. Generational Patterns of Discipline

#### 3.4.1. Corporal Punishment as Commonplace

A majority (*n* = 7) of the clinicians discussed the use of corporal punishment among Black families. Black clinicians noted that receiving “spankings”, “whoopings”, and “beatings” is common within the Black community. Because of this, some clinicians experienced challenges sharing the PCIT method of disciplining children with Black families. Black clinicians found it especially challenging to introduce the PCIT time-out procedure as a new form of discipline without potentially undermining families’ current discipline strategies, especially when they entailed the use of corporal punishment. Sade said that she “get[s] the feeling that they [caregivers] don’t want someone to tell them, ‘No, you shouldn’t spank your child’ and ‘quit doing that. Don’t do that anymore’”. She described the following: “I know that other therapists, either where I’ve worked here, or just in general lean more towards that, or have even actually said that to Black families”. Rather than criticizing Black families’ current parenting styles, Sade suggested expanding the knowledge Black families have about different discipline strategies.

Although one of the goals of PCIT is to encourage parents to use other forms of discipline, Black clinicians noted that the subject of discipline should be handled with sensitivity. According to Joan, “I don’t think you can have a conversation about discipline without talking about culture”. Many clinicians found this to be a commonality with Black parents, including Zara, a 36-year-old clinician with 2 years of PCIT experience. When asked how she brings topics related to culture into PCIT, Zara responded with the following: “I’ll say stuff like ‘how grandma used to raise us’, or ‘what happened when we were younger’, and ‘how we were disciplined’”. Other clinicians (*n* = 2) discussed using Socratic questioning with families to gather their experiences both receiving and using corporal punishment. Because disciplinary strategies are inextricably intertwined with culture, Sade described responding nonjudgmentally when families discussed using corporal punishment during treatment:

If a family says something like ‘I had to break down, and I did spank’, I’m like, ‘That’s okay. That’s a tool in your toolbox’. Even though the goal is to shift away from this utilization of discipline, it [shaming families] defeats the purpose. I recognize that is something that’s still, again, very much a go-to method for families, especially for Black families.

Sade explained that when she acknowledges that families reverting to previously used forms of discipline at times is not uncommon, it tends to increase buy-in and rapport with her Black families.

#### 3.4.2. Alternative Discipline Strategies

Many clinicians (*n* = 7) noted that Black families appreciate PCIT providing them with alternatives to corporal punishment. For example, Nia stated, “It’s often a common thing where [caregivers are] saying like, ‘I was spanked. I was whooped or I was beat, and I don’t want to do that. We don’t know what else to do’”. Many clinicians (*n* = 7) noted that the Black families they serve have an earnest desire to change disciplinary practices that have been generationally passed down. PCIT can serve as an avenue for parents to learn alternative discipline strategies to use with their children besides corporal punishment. When asked what is helpful about PCIT for Black families, Nia responded, “I think that you know the trends that I’ve been saying for PCIT is that it’s an option, or giving people an option to kind of what they describe as breaking generational curses [using alternative disciplinary strategies besides corporal punishment]”.

### 3.5. Racial Considerations

#### 3.5.1. Race Concordance with Black Families

All clinicians (*n* = 10) discussed the unique relationships they share with the Black families they serve. Many clinicians reported that their Black clients sought Black clinicians specifically because they wanted a clinician who had first-hand experience with issues having to do with race. Latoya recalled a client who prioritized having a Black clinician:

The client that’s multi-racial, she was like ‘I don’t mean to be rude, but’, she said, ‘Are you Black?’ And I said, ‘Yes’, and she was like, ‘Okay. I just wanted to make sure’, she said, ‘because I wanted a Black therapist…I didn’t want to have to explain why I respond or do the things that I do to someone who doesn’t look like [me]’.

Black clinicians (*n* = 8) also described believing they are able to explain PCIT concepts to Black families more effectively than clinicians of other races. Black clinicians expressed that because they are familiar with Black culture, they can translate and provide culturally relevant rationales for elements of PCIT, like time-out and ignoring, for Black families more easily, as it comes naturally to them. Ayanna recounted an experience she had with a family:

All my [Black] families, including myself, were like, ‘What is dawdling like?’ We don’t use that language. We’ve never used that word’, and I had to kind of tell them that you know, like the definition of what it is like moving slowly, or someone kind of taking their time.

Because Black clinicians share the same racial background as their clients, they (*n* = 4) expressed knowing when and how to translate PCIT language to improve Black families’ understanding. Given Black clients’ preferences to work with Black clinicians, clinicians (*n* = 5) discussed the dearth of Black clinicians who are trained in PCIT and the need for more Black clinicians to become certified.

#### 3.5.2. PCIT Is Eurocentric

Most clinicians (*n* = 8) made reference to how PCIT is tailored to White families. Clinicians (*n* = 3) noted that PCIT was developed by White people and thus lacks cultural sensitivity to Black families. Nia discussed her concern about PCIT’s Eurocentrism.

I don’t love PCIT as a Black clinician. I don’t love it. It feels again. There’s a qualitative piece to it that’s a little bit different than the other interventions in which I’m trying. PCIT honestly feels very White because it is very rooted and based in some White supremacy, some White supremacist ideations that it’s hard to pinpoint.

Some of the clinicians (*n* = 2) acknowledged that the harsh realities Black children go through due to systemic issues and racism were not attended to in PCIT. Namely, clinicians’ (*n* = 2) described their clients believing that a strict parenting style emphasizing discipline with immediate outcomes is needed to keep their children safe. Although PCIT encourages a parenting style that is seemingly not as strict as traditional Black parenting, Nia elaborates on why Black parents are likely to gravitate to a stricter parenting style:

If you [a Black child] go out, and you’re not listening, and I tell you, ‘You come stand next to me’, and you don’t listen—that could be your life. That could put our families in danger, and so that high control was adapted like that [for] survival, and so that gets passed on through generations and generations.

During Joan’s interview, she also mentioned how strict parenting styles can be traced back to Jim Crow and segregation, where the consequences for Black children acting out were more severe and dangerous. Clinicians’ acknowledgement of the safety concerns their families experience due to racism may inform the reluctance of some parents to adopt parenting strategies that may not produce immediate results (e.g., ignoring, time-out) for children’s misbehavior, which could have exacerbated consequences (e.g., suspension, expulsion, physical harm) for Black children in the U.S. This sort of cultural understanding situating Black parenting practices in the broader context within which Black families live helps clinicians to make individual adaptations to better align PCIT with Black families.

### 3.6. Protocol Changes

#### 3.6.1. Clinician Attributes

Clinicians described a number of qualities that they believe help make PCIT more suitable for Black families. Most notably, those qualities include openness (*n* = 8), cultural competency (*n* = 8), and flexibility (*n* = 7). Examples of openness from clinicians include openness to discussing cultural issues, maintaining an open stance, creating an open space to give clients the room to ponder and change, as well as soliciting feedback from families in treatment. Cultural competency refers to when clinicians describe demonstrating cultural humility, but also when clinicians describe respecting and honoring a family’s cultural traditions, which allow the family to feel seen and validated. Lastly, flexibility specifically refers to clinicians’ flexibility in tailoring treatment for Black families.

Additionally, the majority of clinicians’ (*n* = 8) described the importance of supporting Black families. This support can be shown in a myriad way, including supporting them through issues external to treatment or accompanying them to school meetings to provide education on the role of PCIT, as well as normalizing and avoiding shaming families if they are seemingly reverting to previous parenting strategies, such as using corporal punishment.

Finally, a vast majority of the clinicians (*n* = 8) tended to follow their clients’ lead. This included meeting caregivers where they are in treatment and providing examples to help caregivers connect PCIT principles with their own lives. Clinicians tended to employ this technique, especially in response to caregivers who have low motivation to change their current parenting strategies or in response to caregivers that are not convinced regarding the effectiveness of PCIT principles. Clinicians also expressed understanding of the sociocultural and financial stressors a family may be experiencing. As an example, Kamilah described her thought process when one of her families was facing financial issues: “When it comes down to that, you know, I just had to say we’re not doing PCIT that day, because having food is way more important than her kid that’s cutting up in school”. Kamilah’s ability to meet this family where they were is her own way of making PCIT more culturally sensitive.

#### 3.6.2. Suggestions for Protocol Improvements

Almost all the clinicians (*n* = 9) suggested improvements for PCIT that called for more diversity, equity, and inclusion. This included a proposal for diversity cautionary statements, having specialized questions to assess DEI issues, having culturally relevant resources such as videos, handouts, and books, as well as more cultural sensitivity overall in the therapist’s approach. Multiple clinicians (*n = 2*) mentioned increased time to discuss external stressors with the family. Kamilah described how extending check-ins could better support Black families:

They [PCIT International] didn’t leave a lot of leeway for people to be able to talk about their issues. So when you have a session, they [PCIT] say at the beginning, give them two or three minutes to talk about their week, but our people [Black families], they need more than two or three minutes.

Nia and Joan both suggested the manual include cultural questions to probe the family. Nia suggested a more structured way to talk about race and culture in the manual, and Joan stated that the manual could do a better job of encouraging the therapist to “think about your family’s history” and the impact of “living in the place where they live”. Aisha added that the manual should have a cultural considerations section noting how some cultures present to treatment, similar to what the DSM-5 includes.

Some clinicians (*n* = 3) brought up simplifying the language for better comprehension, regardless of caregivers’ socioeconomic class. Ayanna, for example, described having to break down and explain the language to some of her families. She suggested “changing the language on some of the documentation and making it more, not just culturally competent, but just for populations from low economic backgrounds, making the language a little bit more straightforward”.

Additionally, clinicians (*n* = 9) suggested ways to improve the language used in PCIT specifically for Black families. This could include making the language and/or DPICS coding more flexible or more culturally tailored to Black vernaculars. In her interview, Monica suggested “…a DPICS manual for Black people” and Sade also noted a specific need to account for AAVE: “We have to do like Black English vernacular in there. That’s the biggest thing, I think, is looking at ways that we can make exceptions for certain situations”. Zara further elaborated on this point in her interview:

I think that it would be cool if some of like the the words that Black families tend to use, or like the phrases to empower their child to be considered, not neutral talk, but could be considered labeled praise like “my man”, or “that’s right”, you know, instead of it being like unlabeled praise, and they don’t get credit for that somehow pairing enthusiasm with a culturally relevant phrase, I think, should be considered a positive like point.

Further, Monica described that the Black community encompasses a vast array of different cultures, which impacts language and encourages a more intersectional approach to DPICS:

The DPICS coding, making some adjustments there. I think not just racially but also regionally, like. There are certain things that people say in certain places, like when I was in New Orleans, and there’s a whole other language that we’re speaking of words that don’t necessarily fit into these different categories, and just having some flexibility there.

While the current manual does allow for some flexibility in counting neutral idiomatic phrases paired with a gesture as praise, this approach relies on clinician knowledge of certain speaking patterns, sufficient training in cultural flexibility, as well as potential support from trainers or supervisors. Kamilah noted her concern with certain clinicians implementing PCIT in a culturally responsive way, calling for further training and support:

I think that the White therapists need to get better training on how to work with Black people… if you don’t know how Black people function and work, and what their priorities are, you know it’s hard for you to provide a service and know what our people like. If you’ve never experienced the real Black experience—[and] I think they’re getting it now after George Floyd and Black Lives Matter and the systemic racism–they’re finally starting to wake up and see: their life is very different as a Black person than, you know, your White privilege, so I think that [clinicians] should be required to do some training.

Other clinicians also underscored this point regarding barriers to culturally responsive implementation within the limitations of the current manual in their interviews. These barriers included instances where clinicians tried to flexibly implement DPICS coding but were met with resistance from White trainers and supervisors who were not familiar with the meaning or intention of popular phrases in Black vernacular. This experience further underscores the need for better dissemination of positive, culturally responsive changes to the PCIT protocol in regards to language and coding flexibility.

Overall, Black clinicians enumerated a number of strategies across various domains to increase the cultural sensitivity and quality of care received by Black families participating in PCIT treatment. While PCIT is currently used with Black families, the perception of Black clinicians is clear in that there is room for improvement to better serve this population.

## 4. Discussion

Given the limited research on PCIT with Black families, we assessed the perceptions and experiences of Black clinicians with experience using PCIT with Black families. This is the first known study to specifically investigate Black clinicians’ perceptions of the cultural fit of PCIT with Black families. Using thematic analysis, the following themes were generated: lack of cultural misalignment, manualization, barriers to treatment, generational patterns of discipline, racial considerations, and protocol changes. Overall, Black clinicians identified various points of cultural misalignment in providing PCIT with Black families and detailed their treatment modifications and recommendations to improve cultural sensitivity. Our study contributes to the limited research on PCIT and Black families by expanding our knowledge on whether a cultural adaptation of PCIT is needed for Black families and what modifications may be warranted to increase the cultural relevance and sensitivity of the treatment. Our qualitative findings provide context to studies reporting high attrition rates of Black families in PCIT.

Clinicians described multiple areas of cultural misalignment they have observed when treating Black families using PCIT, including lack of cultural sensitivity, treatment phases of PCIT, perceptions of time-out, and concern regarding external judgment. These findings expand our knowledge on issues clinicians believe Black families may encounter with PCIT treatment, particularly in response to the CDI and PDI phases. Specifically, findings provide a window into some Black families’ reluctance to adopt aspects of treatment, including allowing the child to lead the play, ignoring misbehaviors, providing frequent labeled praises, and using effective commands and time-out. Findings add to previous research on Black parents’ perceptions of play therapy [36], and some findings support previous research documenting Black parents’ negative views on the effectiveness of time-out [37] and influence of extended family and community on parenting behaviors [38]. Families’ concerns about treatment recommendations and reactions from their support system may contribute to the high rates of attrition reported in PCIT studies with Black families, given that parents’ negative perceptions of treatment are a greater barrier to engagement in BPTs than logistical barriers [39]. Therefore, improving Black families’ perceptions of the benefit of treatment for their families is key to improving retention.

Participants discussed how PCIT lacks cultural sensitivity due to its rigid manualization, which is unfavorable for Black families’ successful completion. This finding is consistent with previous research, which found that clinicians often perceive EBTs, like PCIT, as too rigidly manualized to permit individualized tailoring of treatment [40]. Thus, EBTs, including PCIT, are not easily translated to diverse cultural groups [40]. A majority of clinicians interviewed discussed attempts at tailoring the treatment for the Black families they work with, especially regarding language and coding. Although the culturally sensitive translation of materials and concepts mirrors other culturally modified aspects of PCIT for Latino, Native American populations, and cultural groups in general [12,14,17], we are not aware of previous modifications to DPICS coding for other U.S.-based racially minoritized populations. Given the higher number of sessions needed to complete PCIT, previous studies on PCIT with Black families have either removed the requirement for meeting skill criteria goals in order to progress to the next stage of treatment [21] or suggested the removal of the skill criterion [22]. Adapting either the coding criteria to better align with AAVE or reconsidering the skill criteria goals may aid in improving retention of Black families.

Clinicians identified the time commitment required by PCIT and logistical considerations as major barriers for Black families completing treatment. This finding is in line with previous research showing that time commitment is a primary reason for attrition, as well as difficulties with childcare and transportation issues [41,42]. Additionally, clinicians shared that financial constraints were a large barrier for Black families, which is consistent with research showing that SES predicts treatment dropout [24]. While Black clinicians in our study described multiple adaptations to reduce the impact of treatment barriers on attrition (e.g., child-care for non-participating children, flexible appointment options), previous research with Black families with low SES has demonstrated that similar logistical supports were insufficient in reducing the high attrition rates in families [22,23]. These findings may indicate that adaptations to address the PCIT requirements, in addition to common treatment barriers, may be needed to reduce Black families’ attrition rates.

Black clinicians identified discussions regarding corporal punishment as an important area for increased cultural sensitivity for PCIT clinicians working with Black families. Given that Black children’s disruptive behavior has an increased likelihood of exacerbated and harmful consequences resulting from systemic racism [43], it is imperative to address corporal punishment within PCIT with the utmost sensitivity and understanding of its historical significance within Black families. Previous research has shown that Black parents endorse the use of corporal punishment at higher rates than White parents [44,45] and view corporal punishment as the discipline strategy yielding the most immediate compliance [46]. Although the preponderance of research has shown negative child outcomes associated with corporal punishment, research findings are mixed for Black children, with some studies finding that corporal punishment is associated with more externalizing behaviors and other studies finding that it is associated with fewer externalizing behaviors [44,45]. It is also important to note that clinicians’ reports of Black parents seeking to learn alternative forms of discipline support previous findings that Black parents use a variety of disciplinary methods with children and tend to favor discussions with children over corporal punishment [47]. Having a nuanced understanding of the literature on disciplinary practices and outcomes in Black families and taking a sensitive approach to discussions of corporal punishment may improve the perceived cultural sensitivity of PCIT with Black families.

Clinicians expressed concerns that PCIT culturally excludes Black families given racial incongruence with providers and the samples utilized to validate the treatment, Eurocentricity of the treatment, and cultural differences in parenting. Notably, a recent study found that 83% of Black caregivers reported it was important to have a mental health provider of the same race and ethnicity because they felt more comfortable working with someone of the same race, perceived that it was easier to build a rapport with their provider, and valued the representation of having a same-race provider for themselves and their children [48]. Furthermore, our finding related to Eurocentricity mirrors anecdotal reports from previous PCIT research with Black families that reported a Black participant believed PCIT represented a “White” parenting method [23]. Taken together, our findings contribute to knowledge regarding possible reasons for Black families’ high levels of attrition from PCIT and identify possible modifications that may enhance treatment engagement and satisfaction. Further, these findings highlight the important role that attention to ethnic-racial socialization practices could play in creating a more culturally responsive treatment protocol for Black families.

While a more in-depth assessment like the one done in My PCIT may be helpful in identifying barriers and providing psychoeducation to overcome those barriers, more may be needed in terms of deep structure change as well as clinician training, education, and competency development to account for the parenting context in which many Black parents operate. One cultural adaptation for Black families with young children noted the importance of embedding consideration for ethnic-racial socialization practices to inform multiple aspects of treatment, including alignment with parenting goals, content delivery, and how Black parents deliver important messages to their children [49,50]. Multiple clinicians in our study discussed navigating parental worry about safety or navigating racism directed toward their child or their family while employing PCIT skills and techniques. Ethnic-racial socialization goals for Black parents may include modeling and communicating messages around safety and coping with bias that are not readily aligned with the skill criteria of standard PCIT. To this end, clinicians engaging with this population must be sufficiently knowledgeable to be flexible enough to provide what is needed to foster necessary buy-in and maintain engagement throughout PCIT. The additional insight required to be culturally responsive to Black families may come through personal experiences, as is the case with many of the clinicians in this study, or through an approach to adaptation that includes identifying necessary components for modification.

### 4.1. Practice Implications

Despite PCIT’s strength in producing significant improvements for families when addressing disruptive behaviors in young children, disparities remain with respect to completion rates among Black families. The qualitative results from this study demonstrate the need and desire for an adaptation of PCIT for Black families, according to Black PCIT clinicians. Based on their qualitative interviews, multiple areas are implicated in order to move forward the process of creating a culturally relevant adaptation.

For one, Black clinicians are already modifying PCIT to try to serve Black families more effectively. These modifications are occurring in multiple ways including modifying DPICS coding and other language used, explicitly addressing concerns about therapeutic interventions, adding more time for PDI preparation and extending check-ins to address external issues, incorporating additional family members into sessions and working through how clients can respond to family and community judgment, addressing discussions regarding discipline and corporal punishment in ways that take into account historical context and present-day racism, bringing in shared identity as means to build trust, as well as validating the difficulty families face in implementing PCIT amid concerns that family and community members may judge their parenting practices. However, without formalizing these modifications as an adaptation that is disseminated in the training of PCIT therapists, these types of changes are used ad hoc based on the cultural competence of clinicians and their familiarity with the Black community. As a result, clinicians are not consistently providing care that is culturally relevant to Black families, which may foster further mistrust in the mental health system. Clinicians in this study also noted barriers to implementation of their desired culturally responsive modifications (e.g., coding a statement as Praise in DPICS) due to perceived rigidity in the manual communicated through their training or pushback from trainers or supervisors who do not have familiarity with Black cultural and language practices.

This data highlights Black clinicians’ perceptions of the current PCIT practices that need modification, not only to best serve Black families but also to support clinicians in delivering this manualized treatment in a culturally sensitive fashion. Clinicians who are providing PCIT in an effective manner are already making changes and exceptions, particularly with regard to language. In this study, Black clinicians highlight that attending to language used in PCIT is a must–both in structure and content. Structurally, the DPICS coding manual does not account for AAVE. Assuming that all families speak in a way that is divorced from cultural differences denies families access to this approach. Informing DPICS with cultural differences in speaking patterns would better match the intended effect of language rather than a blanket dismissal of certain types of speech patterns. While there may be more delineation of some nuance in the DPICS research manual (e.g., idiomatic neutral phrases with gesture coded as commands), many clinicians are trained with the abridged training manual, limiting dissemination efforts. With respect to content, the language used in the treatment manual (e.g., “minding, dawdling”) is outdated and necessitates reconsideration.

Limitations to PCIT for Black families also extend to its emphasis on language as it relates to meeting goal criteria of skills taught during treatment, particularly CDI. Prior editions of the treatment protocol have included the word “mastery” which has since been updated in the 2011 treatment protocol (updated in 2022). However, most clinicians continue to utilize the old treatment manuals with which they were trained. Dissemination of the updates in language (i.e., replacing “mastery” with meeting “goal skill criteria”) has not reached all clinicians, as evidenced by the language used by Black clinicians in this study. Another important change relates to the distinction of trainers. PCIT International has replaced the use of “master trainer” with “global trainer”. However, this language was still utilized within interviews with PCIT clinicians, again demonstrating the need to amplify communication around these important changes. Training updates, such as the use of the words “mastery” and “master” also need to be extended to those who were previously trained on outdated protocols in order to improve inclusivity and reduce harm for Black families, as well as clinicians, that can be caused by this type of language.

The clinicians who participated in this study communicated that PCIT feels more attuned to White families and has been developed by White individuals for White families. That is to say, the gestalt of PCIT is centered around Whiteness. To improve its inclusivity and respond to the needs of those who do not identify as White, in particular Black caregivers, PCIT can improve by better accounting for the experience of being Black in the U.S. For example, consider safety concerns for Black children who are disruptive in public settings or the heightened concerns among Black parents for CPS to be called when allowing for a child to tantrum until extinction as part of the time-out sequence. The different consequences Black families experience based on race, due to racism, cannot be ignored when being sensitive to families’ circumstances and experiences.

Future research in this area should systematize clinicians’ strategies for increasing the cultural sensitivity of PCIT for Black caregivers and test the retention and engagement of Black families receiving the adapted version. In alignment with previous cultural adaptations and best practices, perspectives from Black families regarding their PCIT experience are highly needed to inform possible adaptations.

### 4.2. Limitations

Despite the multiple strengths of the current study, the findings must be interpreted in the context of the study’s limitations. First, while the qualitative data represents rich and strong themes developed across participants, a small sample size may mean that the data were not representative of Black PCIT clinicians’ views broadly. Second, utilizing clinician’s perspectives of Black families’ experiences with PCIT may miss familial experiences that were not shared with or obvious to clinicians. Third, though all clinicians were trained in PCIT, experience levels ranged, including some who had been practicing PCIT for a short number of years or who had utilized PCIT with a small number of Black families. To this point, there was a wide variation in completion rates across our clinicians. Four of the five clinicians reporting the lowest completion rates reported practicing PCIT for under five years, whereas all five clinicians with seven or more years of PCIT experience reported completion rates of at least 50% (*M* = 70%). Fourth, we did not systematically gather the type of setting (e.g., private practice, community-based) in which clinicians saw clients nor the typical SES of the Black families served by the clinicians. The absence of this data may obscure any differences in treatment recommendations as a result of SES. Moreover, this information may provide contextual explanations for the variation in completion rates beyond clinicians’ PCIT experience levels. Fifth, although all clinicians were trained in the standard PCIT protocol, the extent to which clinicians may have been utilizing or incorporating adaptations of PCIT into their work with Black families is unknown and may have contributed to clinicians’ experiences with and perceptions of using PCIT with Black families. Last, the nature of interviewing clinicians over Zoom when pulling from a relatively small PCIT clinician community could have censored clinicians’ expressed thoughts about PCIT.

## 5. Conclusions

This is the first known qualitative investigation of the cultural fit of PCIT with Black families. The current study expands the literature on BPT interventions with ethnically minoritized populations by offering insight into the perceptions of Black clinicians using PCIT with Black families. Additionally, this research furthers understanding of techniques Black clinicians use to make their work with Black clients more culturally acceptable. Black clinicians provided meaningful insights into the experiences of Black families participating in PCIT that may inform the reasons contributing to the high attrition rate of Black families, as well as ways to increase the cultural sensitivity and relevance of PCIT for Black families. This study can inform future research in adapting PCIT for Black families as informed by Black clinicians and their experiences with Black families and determine its impact on acceptability and attrition rates. More specifically, this study highlights potential changes in the implementation of PCIT that may increase the cultural relevance and responsiveness of the intervention for Black families so they may benefit more fully from this robust intervention.

## Figures and Tables

**Table 1:** Participant Characteristics.

Clinician Pseudonym	Title	Gender	Age	Years of PCIT Experience	Number of Black Families Receiving PCIT	Completion Rate
Nia	Independent practitioner	Female	-	14	35	75%
Aisha	Licensed psychologist	Female	32	10	2	100%
Jamila	Clinical social worker	Female	38	3 ½	20	10%
Latoya	Clinical social worker	Female	39	2 ½	3	0%
Ayanna	Professional counselor	Female	35	3	5	40%
Kamilah	Clinical social worker	Female	62	11	30+	50%
Sade	Clinical social worker	Female	38	4	5	20%
Monica	Licensed psychologist	Female	30	7	7	75%
Joan	Clinical psychologist	Female	34	10	35	50%
Zara	Clinical psychologist	Female	36	2	7	90%
Nia	Independent practitioner	Female	-	14	35	75%
Aisha	Licensed psychologist	Female	32	10	2	100%
*M (SD)*			38.2 (9.4)	5.4 (3.6)	15.3 (14.7)	51% (34.1%)

**Table 2:** Interview Questions.

Interview Questions		
Please describe your experience conducting PCIT with Black families		
What was the process like?		
In what ways do you think PCIT was helpful for Black families?		
In what ways do you think it was NOT helpful for Black families?		
Overall, what percent of the Black families you provided PCIT completed treatment?		
Assessment	CDI	PDI
Approximately what percentage of Black families completed the assessment phase of PCIT and continued on to treatment?	Approximately what percent of Black families completed the CDI phase?	Approximately what percent completed the PDI phase?
How do you bring in topics related to cultural background and race into the PCIT assessment?	How do you bring in topics related to cultural background and race into the PCIT CDI phase?	How do you bring in topics related to cultural background and race into the PCIT PDI phase?
Thinking about Black families you worked with who dropped out during or immediately following the assessment phase, why do you think they dropped out?	Thinking about Black families you worked with who dropped out during the CDI phase, why do you think they dropped out?	Thinking about Black families you worked with who dropped out during the PDI phase, why do you think they dropped out?
What changes would you like to see made to the assessment phase to make PCIT more culturally relevant and acceptable for Black families?	What changes would you like to see made to the CDI phase to make PCIT more culturally relevant and acceptable for Black families?	What changes would you like to see made to the PDI phase to make PCIT more culturally relevant and acceptable for Black families?
Are there any other examples of how you bring in topics related to culture and race into PCIT when working with Black families?		
At what point in the assessment and treatment process was there the most dropout with Black families (during assessment or before treatment began, during CDI or before PDI began, or during PDI)?		
What is your hunch about why that point is when the most attrition occurred?		
What feedback have you received from Black families who have participated in PCIT? What worked well? What could be improved?		
What changes do you think are needed to make PCIT more culturally relevant and acceptable for Black families?		
How comfortable do you feel providing PCIT to Black families on a scale of 1–10? (with 10 being the most comfortable)		
How do you feel providing PCIT as a Black Clinician? Are there things that seemed to work well versus not?		
Anything else you’d like to share about your experience providing PCIT to Black families?		

**Table 3:** Summary of the results.

Themes	Subthemes	Supporting Quotes
Cultural Misalignment	Cultural Sensitivity	Let’s have more conversations, let’s do all these things, and it may not be the right fit for treatment. We may need something else for these families. And that is okay. It may be that culturally, like time-out is not going to work.
	Child Directed Interaction (CDI)	That not being something, again within the Black family, that culture that we typically do, you know… children are meant to be seen, not heard, you know, and so to allow ourselves the adults to allow themselves to get on that level and let the kiddo lead.
	Parent Directed Interaction (PDI)	I think PDI is hard for a family…I don’t even recommend PCIT until I’ve had a chance to talk with them about it and talk about the difficulties in PDI, and I do a lot of support in PDI again.
	Time-Out	Truth be told, a lot of Black families really thought that time-out was for White people. I’m like, “What do you mean for White people?” They said “That’s what White people do”… so trying to get Black people to change their philosophy and the way they view things that discipline is discipline. It doesn’t have a color on it.
	External Judgment	I have done one family in particular to Walmart to do our public behavior session, and Mom was super nervous about it, was very worried about it, but we like she did it and did it beautifully, and, like you know, kid was like on their best behavior. But I think that just that there’s already a lot of fear about how you’re perceived, and then taking that to a public space can be intimidating.
Manualization	PCIT Is Too Rigid	I think a lot of our clients were not really that happy with PCIT even after they finished it, because you know the wording. Everything was so rigid. You have to do this, and in this, you know, they need to have a little bit more flexibility.
	Language Concerns	But I found that kind of randomly throughout the process, and the manual of different things. There were other words… that just wasn’t culturally competent. I mean that word could have just been. Does your child take their time or move slowly in between tasks as opposed to ‘dawdling’?
	DPICS Coding Incompatibility	When they [Black families] were struggling with trying to make mastery because, and I think again, it may have been about the language because they wanted to be… It is very specific. You have to have label praises and the behavior descriptions and the reflections.
Barriers to Treatment	Time Constraints	So, we found a lot of time. It’s the commitment, and that they [Black families] want the additional information. They just don’t—they can’t keep up with what we’re asking.
	Socioeconomic Status	A lot of my [Black] families were trying to make ends meet. So, they had to work. Working was a number one thing, and it wasn’t that they didn’t want the service for their child, but they had to work.
Generational Patterns of Discipline	Corporal Punishment is Commonplace	I think… specifically Black families. Not always. Corporal punishment has either been used or is being used, and it can be something that’s like, I think, normative.
	Alternative Discipline Strategies	I think that you know the trends that I’ve been saying for PCIT is that it’s an option, or giving people an option to what they describe as breaking generational curses.
Racial Considerations	Race Concordance with Black Families	I think with the Black families that I did work with, it was successful in a way, I think, because I also am Black, too. So I think we were able to relate to each other when it comes to skills.
	PCIT is Rooted in Eurocentricity	I will say things like PCIT was developed by White people some of the ways in which the language is crafted, and what I’ve been told to observe for doesn’t always apply to our community
Protocol Changes	Clinician Attributes	I’m going to identify my race. I’m going to identify things like how the world sees me, how I see myself, or how I want to show up in the world, right? So, these are two things I think that a lot of clinicians don’t do, especially if they’re not from a marginalized background. I think that in order to create a safe environment a lot of time for some Black families, you need to be transparent about that, right? This whole idea of being color blind or ambiguous is not… It doesn’t usually sit well with our community from my experience.
	Suggestions for Protocol Improvements	Giving people a little bit more guidance about how to bring in race and culture… giving some guiding language or exposure to that… [In] my manual, they lean a lot on research like oh, you know, I can talk about the resources. Not to say all, but a lot of the Black families don’t really care about the research, because historically the research has either not included us, or we’ve been experimented on. And then the intervention was never applied to us, right? And so sometimes you say, ‘well the research says’, or like you relate to research, that can actually create additional barriers for families. And so, I think that the manual doesn’t always do it… doesn’t do a really good job of bringing up race and culture and giving people that structure and guidance, because, remembering that clinicians are gonna take this manualized thing and say, ‘I’m gonna like read this like my Bible, and I’m gonna go forward and do it’. That doesn’t mean they have any other actual external skills. We would hope so, but a lot of them don’t have the external skills to be able to have these conversations, so PCIT could do better in supporting that.

## Data Availability

The data presented in this study are available on request from the corresponding author. The data are not publicly available due to privacy.

## Appendix A

**Table A1:** Qualitative Codebook regarding Black Families Experiences with PCIT-Clinician Version (Version Date: 31 July 2024) [33].

Themes	Sub Categories	Unique Codes	Abbreviated Codes
Barriers to Treatment Attendance	Barriers	More Barriers/Obstacles Generally	BARRIERS-G
		COVID presented challenges in terms of PCIT implementation with clients	BARRIERS-COVID
	Time Constraints	Barriers—Time Constraints (Rammy)/Time Sensitive—Competing Responsibilities to Navigate—GENERAL	TC-G
		Feasible	TC-FEASIBLE
		No time to consistently come to therapy	TC-TX ATD
		Competing work responsibilities/Work Schedule /Job Loss or Threat of Job Loss)	TC-JOB
		Competing other children’s needs	TC-O-CHILD
		Sessions were time-consuming	TC-SESSION-L
		No time to get daily homework in	TC-HWK
		PCIT takes too long; Or is too much work; or too much time commitment	TC-PCIT
		Achieving Mastery is difficult and takes too Long/Mastery Takes too Long	TC-MAST-DIFF
		CDI takes too long to master; family get tired and frustrated that they can’t move on to PDI	TC-MAST-CDI
		PDI takes too long to master; family get tired and frustrated that they can’t graduate from treatment	TC-MAST-PDI
		PCIT vs. Other Therapy (Greater Time Commitment)	TC-PCITvs-O-TX
		School Disruption for Therapy Sessions	TC-SCHOOL
		Time-Out inconvenient w/regard to time	TC-TO
	Stress/Crisis	Crisis: Aggregated Stress Severity and Family Dysfunction (SES-Financial Stress; job eviction) family in crisis Parental stress and life events)	CRISIS
		The Evaluative/Assessment Component of PCIT feels Stressful/Judgmental	PCIT-JUDGE
		PCIT is difficult and Stressful—family is frustrated with the treatment PCIT is Stressful	PCIT-STRESS-DIFF
	SES	SES: Socioeconomic disadvantage/Financial Constraints (Eviction; no food; job loss)	SES-G
		SES- Middle Class families have more resources that enable them to do better in PCIT treatment in which they adapt to the guidelines, make changes a little bit quicker, and tend to be more consistent	SES-MIDDLE
		Transportation	SES-T
		Insurance	SES-I
		Family does not have the money for appropriate PCIT toys; Family may have inappropriate toys	SES-INAP-TOYS
		Difficult living circumstances (Housing)	SES-H
	Attrition	Pre-Screen	ATR-PRE-SCREEN
		Assessment Phase (specify direction—e.g., if clinician notes drop, “decrease”)	ATR-ASS
		CDI Phase (specify direction—e.g., if clinician notes drop, “decrease”)	ATR-CDI
		PDI Phase (specify direction—e.g., if clinician notes drop, “decrease”)	ATR-PDI
		Losing one of the Parents (ex: work schedules, childcare issues)	ATR-PAR
		Don’t Make it to Graduation	ATR-GRAD
		Attrition—GEN	ATR
	Attendance	Infrequent or inconsistent Attendance	IFR-ATTEND
Related Treatment Issues (Could be neutral, negative, or beneficial)	Coaching	PCIT is intensive INVIVO coaching	PCIT-COACH-INTENSIVE
	Referral Source	Family/SELF VS. OTHER PROFESSIONALS	REFR
	Forensic	Court Involvement	COURT
	Research	PCIT is Data Driven (e.g., ECBI) (Now we have this code here under barriers but sounds like it could be positive or negative)	PCIT-DATA
		PCIT is too Research Focused	RESEARCH
		PCIT Research needs to be ethnically diverse as well	PCIT-RES-Needs-Diversity
Manualization/Rigidity	General Rigidity	PCIT is Rigid but a desire to ensure fidelity which seem in opposition of cultural sensitivity	RIG-PCIT
	Language	Language is Problem/Dated/Awkward/Inappropriate	LANG-DATED-AWK
		Language—Socio-Economic Status—Family Doesn’t Understand (e.g., words too big)	LANG-SES
		Language is Too Rigid	LANG-RIG
	Too Manualized	Manualized/Structured (neutral)	VERY-MANUAL
		Too/Very Manualized/Too Manualized (more negative)	TOO-MANUAL→VERY-MANUAL
	Mastery Time	Rigidity of Mastery Criteria	RIG-MAST
	Rigidity of Weekly Check Ins	PCIT too Rigid (e.g., TIME DURATION: only 2–3 min to process crisis when need more; 14-weeks is a lot)	RIG-CHKIN
	Flexibility	Reminders of where there is flexibility in PCIT (e.g., Public Behaviorsis often treated as required when it is an optional session)	PCIT-FLEX
	PDI	PDI is Too Rigid	RIG-PDI
	Method of Teaching PCIT to Caregivers	Method teaching PCIT to caregivers is important to consider: Want to make sure not to be Condescending—E.g., with the use of Powerpoints	PCIT-METHOD
	DPICS	DPICS Coding is too Rigid	RIG-DPICS
	Paperwork	High demand of paperwork for treatment (Qualifies for Homework Sheet, ECBI sheet, etc.)	HI-P-WRK
Infrastructure	PCIT	Poor Home Infrastructure: No Private Space to practice PCIT at home	INFR-PCIT
	Agency	Cultural Competency	INFR-AGENCY-CC
		Agency has the all the necessary elements for conducting PCIT’s specialized tx; one-way mirror; bug-in-the-ear.	INFR-AGENCY-PCIT
		Solicits Feedback from Family on Treatment	INFR-AGENCY-FEED
		Agency provides supplements to PCIT tx such as transportation vouchers, childcare	INFR-AGENCY-TX-SUPLMTS
		Need for Additional Agency Resources to Support PCIT within their Agency	INFR-AGENCY-RES
	Time-Out	Poor Home Infrastructure: Time-Out Room	INFR-TO
	Session content and time allocation	Working with Black Families PCIT Takes Longer	INFR-MORE TIME
		Need for increased processing time to discuss things going on in their lives	INFR-MORE PROCESS TIME
	Location	Clinic Based	LOC-CLINIC
		Telehealth	LOC-TELE
		In-Community	LOC-CMTY
		Home Based	LOC-HBS
		Issues related to school involvement	SCHOOL
Family Constellations		General	FC-G
		Single parent households	FC-S
		Lots of children in the home	FC-CH
		Grandparents are caregivers	FC-GRAND
		Different types of caregivers ex. Aunt and Grandma	FC-EXT-CG
		Divorced or separated parents (time sharing confusion and differences in view of tx and parenting)	FC-D/S
		Foster parents	FC-FOSTER
		Multigenerational parenting support (PCIT Therapist must understand the important role they play below)—Non-Fictive Kinship Networks	FC-MGS
		Multisystemic parenting support (PCIT Therapist must understand the important role they play below)—Fictive Kinship Networks	FC-MSS
		There’s No Family Support—GEN	FC-FAM-NO-SUPPORT
		Including other family members into treatment to improve buy-in and support primary caregiver during treatment	FC-FAM-SUPPORT
Child Bx Severity		Children resistant (ex: coming to tx, rejecting the therapy/parent)	CH-BX-RES
		Clinician makes reference to child behavior that doesn’t classify as severe	CH-BX
		Severity of child externalizing problems (e.g., hitting parents)	CH-BX-SV
Parental Attributes		Conflict b/w the Parents	P-CONFLICT
		Parental Stress	P-STRESS
		Parental Fatigue	P-FATIGUE
		Parents were Adept at Picking Up PCIT Skills	P-PROFICIENCY
		Black Families want Help and Want PCIT	FAM-HELP-PCIT
		Black family does not offer much feedback regarding the treatment	FAM-TX-SILENCE
		Parent psychopathology (Depression, Anxiety, etc., except for Trauma which is scored below under generational curses)	P-PSYCHO
		Clinician does preliminary assessment of the general family fit with PCIT in terms of general screeners- Family GOOD FIT. Clinician talks about how the structure of PCIT was a bad fit for the family	FAM-PCIT-FIT-GOOD
		Clinician does preliminary assessment of general family fit with PCIT in terms of general screeners—Family BAD FIT: Clinician talks about how the structure of PCIT was a bad fit for the family	FAM-PCIT-FIT-BAD
		Caregivers feel the focus of therapy should be on child rather than parent	P-child-focus-not-parent
		Parental/family member Incarceration	P-JAIL
		Death/loss of caregiver of family member	P-DEATH
		Caregiver has severe medical illness	P-ILLNESS
Trauma and generational curses/Seeking change		Legacy of Generational Curses; parenting traditions; Not necessarily Traumatic.	GC
		General trauma	GC-TRAUMA-G
		Racial Trauma	GC-RACIAL-TRAUMA
		Generational Trauma from discipline	GC-TRAUMA-IG-DISCIPLIN
		Generational Trauma from discipline (Corporal Punishment)	GC-TRAUMA-IG-CP
		PCIT is an option for breaking those generational curses	GC-PCIT-TX
		Parenting Styles Authoritative	GC-Authoritative
		Parenting Styles Authoritarian	GC-Authoritarian
		Black parents are rigid and set in their ways regarding their parenting styles	GC-BLK-PARENT-RIGID
Cultural Misalignment	Race	Black Racial group differences/Clinician makes comment specific to Black families	CM-RACE
	Racial Socialization	Need to nurture relationship bonds between BLK parent and child despite discipline to prepare to deal with being a Black child in an oppressive system where they may be the minority.	RS-PC-BONDING
		Racial Socialization: Using the PRIDE Skills to Foster Black Cultural Pride; And Cultural Rituals (ex: combing hair)	RS-BLK-PRIDE
		Black Parents having “The Talk” w/their Black Children	RS-THE TALK
	Time-Out	Time-Out (=ABUSE)	CM-TO = ABUSE
		Time-Out won’t work (Won’t work; child won’t stay in the chair); Parents may feel like TO allows the child to escape listening to what they’re told to do; or the parents have cultural values that go against the use of time-out	CM-TO
		Time-Out is stressful; the process is stressful for either the family, the clinician, or both	CM-TO-STRESS
	Ignoring	Active Ignoring/Differential attention	CM-IGNORE
	Disability	Disability	CM-DIS
	Complex Intersectionality	Complexity of Ethnic/Racial Identity Intersecting with Other Multiple Levels of Diversity; There is heterogeneity among Black People so there is not a one size fits all Blacks.	CM-INTERSECT-COMPLEX
	Discipline	Discipline—separate out cultural issues of discipline with no mention of trauma	CM-DISCIPLINE
	Corporal Punishment	Use of Corporal Punishment	CM-CP
	Talk	Clinicians report that Black families tend to focus on teaching as opposed to playing during CDI; there is a value/goal for educating their children	CM-TA
	Label Praise	Whether or Not Parents Should Praise Their Children; Caregivers feeling like they are being fake or struggle w/feeling disingenuous or not authentic by praising their children all the time, for no reason; it does not feel natural	CM-LP
	Command	Families want their kid to do what they’re told; EXPLANATIONS ARE CULTURALLY RELEVANT SO IT MATTERS NOT IF IT IS PROVIDED BEFORE THE COMMAND OR AFTER; VARIABILITY IN WHAT IS A NORMAL TONE OF VOICE.	CM-CO
	Enthusiasm	For Black Families “E” culturally has a wide variety of expressions that may not necessarily be the same (e.g., banter/teasing) or captured; although “E” is a PRIDE skill, it doesn’t get credit; needing to distinguish gesture vs. enthusiastic expression. Therefore, looking at child’s cues for how they interpret the behavior either as praise or negative talk should be taken into consideration	CM-E
	CDI	CDI—Letting Child Lead or playtime feels like a luxury; taking issue with special time or use of several of the PRIDE skills	CM-CDI
	Special Time	Special Time	CM-ST-HWK
	PDI	PDI is challenging b/c it goes against cultural traditions, and Blk parents may be resistant to change vs. the actual task of PDI is complicated or difficult to learn and implement	CM-PDI
	Graduation	Parents take what they need and don’t feel the need to hit all the milestones of graduation before terminating	CM-GRAD
		White Families tend to graduate more than POC families	CM-WHT-GRAD
		White families were difficult and or rigid to work with in PCIT tx	CM-WHT-DIFF
		Families that tended to graduate had more resources and were from 2 parent households	CM-GRAD-RESOURCES
	Public Behavior	Public Behaviors—Discomfort and Vulnerability	CM-PB-VUL
		Public Behaviors—Fear of neg interaction with Police Threat—Police are actually present/involved	CM-PB-POL-INT
		Public Behaviors—Public Scrutiny of general public re: parenting→e.g., Police being called	CM-PB-SCR
		Public Behaviors—Public Scrutiny within Black Community	CM-PB-BLKC
	Extended Family Judging	Extended Family/Blk community Scrutiny; Other Family Members will Use Other Disciplinary Strategies Not Consistent with PCIT	CM-EX-FAM-JUDGE
		Need for BLK PCIT Peer Support Group. Perhaps those how have gone through PCIT successful to help those coming along and who will understand what they are going through in trying to break generational patterns	CM-BK-PCIT PEER-SUPPORTS
	Respect Violations—Cultural Taboo	Taboo To Disclose Family Secrets	CM-FAMILY-SECRETS
		Crossing Taboo Lines of respect of elders/Cultural Norms regarding RESPECT	CM-DISREPECT-CUL
	Cultural Sensitivity	Need for Cultural Sensitivity	CM-CUL-SEN
		Use Theoretical principles (such as behavioral principles, coercive cycle; Baumrind’s principles) to explain cultural nuances of Black culture; parenting; families.	CM-THEORY
		PCIT is Cookie Cutter—One size fits all	CM-ONE-SIZE
		General Misalignment in Language	CM-LANG
		PCIT Has been translated into several different languages	PCIT-DIVERSE-LANG
		Need for Cultural Sensitivity-Recognizing PCIT community calling for change in recent years	CM-CUL-SEN-CALL-CHNG
	Family Adherence Despite Opposition	Black Families go along with PCIT even though they Don’t Agree with the Principles	CM-OPP-PCIT-ADHERE
	Cultural Skepticism/Mistrust—Coming to Jesus Convincing	Mistrust—GEN	CM-MISTRUST-G
		Research	CM-MISTRUST-RESEARCH
		PCIT	CM-MISTRUST-PCIT
		Mental Health—A reluctance to seek mental health services or to go outside the family to ask for help; Mistrust of Mental Health Services to provide good and ethical care to POCs.	CM-MISTRUST-MH
		Need to Create a Safe Environment in PCIT where families don’t feel judged by clinician/treatment; also to capture notions of keeping the family safe with PCIT interventions; Elaborated on this Definition to include making sure tx is safe for the blk family: family feels safe to be their true and authentic selves and candidly share with the clinician.	CM-NEED-SAFETY
	Expectations	Misalignment of Parental Expectations of the PCIT Treatment; Or unrealistic or inappropriate expectations re normative child behavior (e.g., expecting child to be perfect all the time.)	CM-FAM-EXPECT
		Perception That PCIT Expects Clients to Achieve the Goal of Perfect Parent	CM-EXPECT-PER-PAR
		In order to expect optimal treatment, clinicians tells parents they have to be involved and/or consistency; parent feels too much focus is on them rather than their child	CM-PCIT-EXPECT
	Urgency	Urgency for management of child’s misbehavior; getting kicked out of schools for their behaviors	CM-URGENCY
Systemic racism/Discrimination and PCIT	General Systemic Racism/DIS	Systemic Racism Discrimination	SRD-G
		Mention of BLM and/or George Floyd and impact on PCIT	SRD-BLM
		PCIT needs to be more accessible to Black/marginalized groups	SRD-TX-ACCESSIBILITY
	Inadequate Preparation	Is inadequate preparation for Black child for racism/discrimination they’re going to face in the future; racial socialization	RAC-INAD-CH-FUT
	Racist Language/Terminology	PCIT Language and terminology is racist and/or Eurocentric	RAC-LANG/TERM
	Very Eurocentric/White	PCIT is Very White	RAC-VWHITE
	Clinician Training/Honoring Black Parents	Black Clinicians Fear Consequence for Speaking a Difference of Opinion or Challenging the Model in a Eurocentric Training Model/Honoring of the Historical Legacy of Effective Parenting Among Black Caregivers for BOTH Black and White Children for Centuries	RAC-BLK-CLIN-SILENTRAC-BLK-CRGR-GOOD
	Threatens Cultural Values	feel like parenting style is being threatened—invalidation of self as a parent and potentially cultural norms re parenting; some fear possibly about how that might be perceived→attrition. Tells them to change their discipline strategy that infers they are bad parents?	RAC-CVT-INVAL/BP
Assessment/Incompatible DPICS Coding: Giving Credit where Credit is Due	General	Clinician mentions DPICS coding	DPC-GEN
	Difficult	PDI is difficult to code	DPC-PDI-DIFF-CODE
	Awkward and Too Specific	awkward: RF and BD—Don’t normally speak like that	DPC-AWK-RF/BD
		Too Specific	DPC-SPECIFIC
	Language	Too Much Emphasis on Language for Mastery; DPICS Mastery is too Language Based (e.g.: Parents are Demonstrating Nurturing Behaviors, but they just aren’t using the Language; and in some instances it may be a cultural misalignment particularly for Black fathers)	DPC-LANG
	Vagueness	There are Areas Where Vagueness is Challenging	DPC-VAGUE
	AAVE	Referenced to African American Vernacular English and uncertainty in how to code it	DPC-AAVE
	Praise vs. LP vs. NTA vs. Command	Praise vs. LP	DPC-LP
		Praise vs. NTA; Cultural Directness of Speaking that May not be Considered Negative Talk	DPC-NTA
		Praise vs. Command (“Look at You”)	DPC-CO
	Questions and Command Frequency	Blk Parents ask lots of questions and give lots of commands	DPC-QU/CO
	Assessment	Assessment #’s seem incongruent with feedback and qualifying the nature of the Parent–child relationship	ASSESS-INCONGRUENT
Race Matching—Bilingual/Bicultural	Race Matching w/Blk Families	Black clinician uses insider knowledge about the Black culture w/Black Clients	RM-BIL-Insdr-knowledge
		Need for Racially Matched Clinicians to work w/Black families clinically and in research (Code Switching)	RM-BIL
		Racial/ethnic matching→easier building of trust and rapport between therapist and client	RM-TRUST/RAP
		Racial/ethnic matching increases the blk family engagement in tx	RM-BLK-FAM-STAY-in-TX
		Racial matching between clinician and client(s) intersecting w/gender	RM-BIL-GEND
		Black Therapist is Bilingual (dg: Code Switching)—Coding DPICS	RM-BIL-COD
		Just b/c they share the same race/Clinician tries not to generalize: Black culture is heterogeneous and complex; it’s not just one thing.	RM-BIL-NO Generalize
		Black Therapist is Bicultural (Code Switching)—PCIT Concepts effective explanation e.g., TO and Ignoring—Emphasizing Adding Parental Resources (Tools/Toolbox) Rather than Replacement; How to use the PRIDE skills to promote their value in educating their children	RM-BIL-EXP
	Biculturalism	Therapists use Biculturalism/Bilingualism—GEN (Code Switching) (Not related to race)	BICULT-GEN
	Black Clinician Bilingual Coding	Coding for Family vs. Supervisor to get certified	RM-BIL-COD-SUP
	Toys—Race Matching	Race Matching—Toys	RM-BIL-TOYS
	Therapists	White Therapist don’t understand	RM-BIL-WHT
		Black family challenges the credibility of the blk clinician for using PCIT which they deem to be a white tx with them as the Black family	RM-Blk-FAM-Chlg-Blk-Clin
		Didn’t do Bicultural Translation	RM-BIL-NOTRANS
		Black Clinician DOES feel like PCIT is a Good Fit; PCIT works with Black families	RM-BLK-CLIN-WORK
		Black Clinician feels like doing PCIT with Black families is a way of giving back to the community.	BLK-PCIT-GBack
		Black Clinician DOESN’T feel like PCIT is a Good Fit	RM-BLK-CLIN-NO-WORK
		Believe Other Treatments Work Better for Black Clinicians/Families	RM-O-TX-R-BTR
Receptivity of Black Families to Psychoeducation	Receptivity	Receptivity of Psychoeducation of Blk Families (explaining TO and PCIT has no color; trying to get Blk families to change their philosophies around discipline) Including Come to Jesus sermons to motivate families to move forward with PCIT if they are skeptical	PSYCHO-ED-RECEP
	Humor Approach	Use of humor to build rapport	PSYCHO-ED-HUMOR
	Understanding TX Relevance	Not understanding the relevance of treatment	PSYCHO-ED-TX REL
		UNFAMILIARITY TO PCIT TERMINOLOGY and CONCEPTS	PSYCHO-ED-TERM/CONCP
Suggestions	Funding	General Need for funding for PCIT in Agencies	SUG-NEED-FUND
	General	General Suggestions	SUG-GEN
	School	Need for Incorporation of PCIT in the School Environment in Collaboration with Child Being in Treatment with their Families; Clinician may accompany them to school meetings with team IP planners, teacher, administrators to explain PCIT and what the family is doing in treatment; or consult w/teachers in managing classroom behaviors	SUG-PCIT-SCHOOL
	Dissemination/Buy-In	PCIT Negatively Associated with Problem Children Rather Than a Strength-Based Model that Supplies Parents with A Lot of Useful Tools	SUG-PCIT-P-CHILD
		Clinician States Increased Need for PCIT Dissemination and Buy-In Globally	SUG-PCIT-DISSEM
		Increase Buy-In/Notability Among AA Community (More Publicity); Get the word out about PCIT with the Black community	SUG-AA-BUY-IN
	No Recommendations	No Recommendations—GEN	SUG-NORECOMD-GEN
		No Recommendations—Assessment	SUG-NORECOMD-ASSES
		No Recommendations—CDI	SUG-NORECOMD-CDI
		No Recommendations—PDI	SUG-NORECOMD-PDI
	Trainers	Trainers/Admin/Positions of power need to be Racially Representative	SUG-NEED-POC-TRAINERS
	Clinician Training	Better avenue for training racially marginalized clinicians	SUG-NEED-POC-CLIN
	Black Clinicians	Need more clinicians who specifically work with Black families. This could be Black but also other race clinicians as well; (Such notions go with the clogged pipeline literature.)	SUG-NEED-CLIN-SRV-BLK
	Manual Improvements	Make a list of alternate culturally appropriate toys (boxes, containers, pots, pans, spoons)	SUG-MI-TOYS
		Call for DEI Adaption—Proposal for a Diversity Cautionary Statement and specialized questions—Need for Cultural Sensitivity (not just in manual); Having culturally relevant resources (ex: videos, handouts, books)	SUG-MI-DEI
		Need for improvements re: Language Consideration and/or flexibility with language and/or DPICS; people making recommendations for DPICS coding flexibility; OR breaking down some of the wordy instructions in the TEACH sessions so they are more understandable	SUG-LANG
		Incorporation of greater time PREP for CDI; May involve some problem solving with families	SUG-MI-CDI-PREP
		Incorporation of greater PREP for PDI	SUG-MI-PDI-PREP
		Incorporation of structured processing of TO procedure when TO is long and difficult—e.g., repair of relationship with long tantrum	SUG-MI-POST-TO-PROCESSING
		Remove or make changes to the use of the ECBI	SUG-NO-ECBI
		Include cautionary Statement: leaving room for Flexibility	SUG-MI-FLEX
		Provide information about PCIT early, including some of the infrastructural information and discussing some of the more challenging elements such as TO	SUG-Provide-INFO Early
PCIT International	PCIT International	PCIT International making small steps to change	BEN-PCIT-Intl-CHANGE
CARE and Other Tx Supplements and PCIT Training Models CARE and Other Tx Supplements and PCIT Training Models	Siblings	PCIT sibling session tailoring	PCIT-SIBL
	CARES	Any mention of CARE	CARE
	Gentle Parenting	GENTLE Parenting is Trendy now and Clinician associates it w/PCIT	GEN-PARENTING
	Trauma Focused PCIT Adaptation	Adapt PCIT to have more of a trauma focus	PCIT-ADAPT-TRAUMA
	IOWA-PCIT	IOWA-PCIT which is Attachment Based (Beth Troutman)	IOWA-PCIT
	UC Davis PCIT	UC Davis PCIT	UCD-PCIT
	Chris Campbell—PCIT	Chris Campbell—PCIT	CC-PCIT
	McNeil Teach	Dr. McNeil Teach Powerpoints with Cultural Icon Touchpoint	CLIN-ADAPT-MCNEIL-PP
	Toddlers	PCIT Toddlers	TODDLER-PCIT
	Older Child	PCIT Older Child Protocol	Older Child-PCIT
	CALM	PCIT Adaptation for Anxiety	CALM-PCIT
	GANA	PCIT Tailoring Towards Mexican Americans	GANA-PCIT
	MY	Cultural Adaptation of PCIT	MY-PCIT
	Selective Mutism	Selective Mutism	SM-PCIT
Benefits		Home Based Services	BEN-HBS
		Structure	BEN-PCIT-STRU
		Subsidized services are beneficial because PCIT is expensive	BEN-FUND
		PCIT is effective for most types of families	BEN-PCIT-GEN
		Clinicians found CDI skills beneficial (e.g., improved PRIDE skills of LP, BD, and RF)	BEN-CDI
		Family was able to see the benefits of Ignoring/differential attention for reducing neg to behaviors; and realizing how they may have inadvertently been reinforcing neg child behaviors	BEN-IGNR
		Clinicians found PDI skills beneficial; Or felt PDI was easier to implement compared to CDI	BEN-PDI
		Swoop-N-go is a viable option to TO	BEN-SWOOP
		Improved Parent–Child Relationship Building Due to PCIT	BEN-PCIT-REL
		Behavior—clinicians either believe that PCIT benefits the family and behavior change or commented that it created behavior change in the family; Emphasizing Adding Parental Resources (Tools/Toolbox) Rather than Replacement (unrelated to race matching)	BEN-PCIT-BEV
		Consequences; Discipline	BEN-PCIT-CON
		Bringing Unity To the Families (parents AND children included in the definition of family). Including reunification in court mandated cases of PCIT	BEN-FAM-UNITY
		Parents want Information about PCIT; PCIT information provides validation for the families (the family was already engaging in behaviors that were consistent with PCIT); helps change the way they think about parenting and their children’s behaviors	BEN-INFO
		PCIT is effective w/Black Families	BEN-PCIT-BF
		Black Families Successfully Graduate	BEN-BLK-GRAD
		Family was successful with PCIT	FAM-SUCCESS-PCIT
		PCIT help Parents have a different view and understanding of their children’s behaviors; Possibly seeing them in a more positive light	BEN-PCIT- Teach Parent—Understand Child Behaviors
		PCIT help Parents see the impact of Caregiver’s own behaviors as modeling behaviors for their children	Ben-PCIT-Teach Parent Model
		PCIT Empowers families; Use the skills to uplift and support the family	BEN-PCIT-EMPOWER
		Culturally adapted PCIT is beneficial (clinician employs a culturally humble/open stance)	BEN-CLIN-CS
Miscellaneous	Needs Code	These are items for which there is not already an established code, and we will need a code for	NC
	Can’t Code	We could not understand the point they were trying to make	CD
	Not Applicable	These are irrelevant lines that have no bearing on data collection	NA
	Audio Review	Because the written transcription didn’t make sense, we would need to call for an audio review	AR
	Don’t Know	Clinician didn’t have an answer to the question’ “AKA I don’t know”.	IDK
Clinician Attributes	Clinician	Clinician queries Family regarding their attitudes about treatment. May use PCIT’s “Therapy Attitudes Inventory”.	CLIN-TAI-FEEDBACK
		Clinician extends the times of check-ins to query how things are going for the family outside of PCIT. How are the caregivers doing outside of the child; determining if there are other resources needed or other things worthy of addressing	CLIN-EXTEND-CHKINS
		Therapist Openness to Discussing Cultural Issues/Open Stance/Therapist Openness to Discussing Cultural Issues Maintaining an Open/Respectful Stance; creating an open space to give folks the room to ponder and change.; Solicits feedback from Families re PCIT and Tx	CLIN-C-OPEN
		Clinician did not like PCIT	CLIN-DISLIKE-PCIT
		Honesty, transparency, and directness; calling out the pink elephant in the room that may be serving as a barrier to treatment where the family may be resistant.	CLIN-C-H/D
		Flexibility in tailoring tx	CLIN-C-FLEX
		Cultural Competency/Cultural Humility; Respecting and Honoring the family’s cultural traditions; allowing them to feel seen and validated	CLIN-C-COM
		Clinician temporarily suspends PCIT when other important issues take precedence for the family	CLIN-PCIT-SUSPEND
		Feel Confident using PCIT—Generally	CLIN-C-PCIT
		Feel Confident using PCIT with Black Families or other diverse populations	CLIN-C-PCIT-BLK
		Blk Clinician efficacy w/non-Black Families (ex: how Blk Clin brings their Blk culture to the therapeutic frame when implementing PCIT and how it lands for non-Blk clients)	BCLIN-EFFIC-NON-BLK FAM
		Like doing PCIT with Black Families	CLIN-PCIT-BLK-FAM
		Clinician wants to work with more Black families	CLIN-More-BLK-FAM
		Feels PCIT matches their personality and Clinical style; Or allows for the flexibility to be who they authentically are as a clinician	CLIN-PCIT-MATCH
		PCIT feels like a mismatch for Blk Clinician (e.g., could include the way they speak in use of AAVE; and PCIT terminology; PCIT Lang)	BKCLN-PCIT-MISMATCH
		Making PCIT their own	CLIN-ADAPT-OWN
		Cultural Incompetency	CLIN-C-INCOM
		Clinician finds it difficult to integrate culture into PCIT	CLIN-C-InCOM-PCIT
		Clinician Feels Conflict in the Appropriateness in PCIT’s Application to the Family	CLIN-C-CONFLICT
		PCIT Experience—Low; and may stick more closely to the manual; strictly adheres to model fidelity	CLIN-C-EXP-LOW
		PCIT Experience—High: More experienced clinicians are more comfortable exercising clinical judgment and using flexibility with the therapy model; and exercise less strict adherence to model fidelity; or know how to be flexible without compromising model fidelity.	CLIN-C-EXP-HIGH
		Clinician reports having diverse experiences disseminating PCIT to diverse Families	CLIN-C-DIVERSE-FAM-EXP
		Probing for Cultural Values and History	CLIN-C-PROB-CUL-VALUES
		Cultural conversations come up with general PCIT procedures and Check-ins: E.G. How was your week? Is there anything you want to share or talk about?; Cultural Issues come up during the regular structured check-ins	CLIN-C-GEN-PROC
		Generally likes doing PCIT very much—Reports having a positive experience w/PCIT. Clinician feels that PCIT is a positive mental health experience	CLIN-LKS-PCIT
		Clinician Buys into PCIT; and may use it in their own lives/Uses in Own Life	CLIN-C-USES-PCIT
		Clinician Uses Higher Order Coaching to Draw Connection Between PCIT Skills and Family Improvement	CLIN-C-HO
		Clinician uses elements of PCIT in other treatment modalities when working with clients	CLIN-PCIT-In-O-TX
		Families Trust and View the Clinicians as the Parenting Expert	CLIN-EXPERT-FAM
		Establish Rapport/Trust With Family—What we want to build with the family	CLIN-C-RAPPORT
		Clinician Tries to Use Motivation Interview/Socratic Questions or Other Strategies to Create a Change in Attitude About Cultural Disciplinary Practices in Favor of Learning or Accepting PCIT Strategies	CLIN-CHANGE-FAM
		Clinician finds PCIT hard, difficult, or challenging to implement—clinicians feeling pressure to quickly acquire the skills to implement independently—“I don’t know what I’m doing”/Not Comfortable Doing PCIT Independently	CLIN-PCIT-DIFF
		Clinician Follows Parents’ Lead/Meeting Them Where They Are/Providing Examples to Help Map Onto Lives—IN Response to Parents who Don’t Feel Anything is wrong with the way they parent (ex: CP); or are not completely sold on PCIT techniques.	CLIN-C-FOLLOW-CLIENT-LEAD
		Family is the expert in their lives (the clinician supports the family being the expert in their own lives) AND Clinician employs a spirit of collaboration and partnership; Elaborated on Clin-C-Expert being a collaborative partnering w/the client who is an expert in their own lives; honoring them and leaving them w/their dignity and good intentions in navigating their challenging lives and circumstances that can accompany being Black in America. Honoring the family’s cultural traditions and leaving space for them to incorporate it into the treatment.	CLIN-C-FAM-EXPERT
		Clinician models PCIT skills; Uses parallel process in the coaching	CLIN-C-MOD-PCIT
		Clinician feels pressure to do right by Black families in terms of cultural sensitivity while also maintaining tx protocol integrity. Clinician feels more pressure to ensure that black families get the best ethical treatment.	CLIN-SELF-PRESSURE
		Clinician did not see family to graduation because left agency	CLIN-DK-BLK-GRAD
		Provide additional support to the family; Normalizing the process for the family—Parents who are Trying to Change and Feel Guilty When They Backslide—what we are trying to offer the family—Normalizing and Not Shaming; Supporting them in issues that happen outside of treatment as they join them in their journey of meeting them where they are.; e.g., accompany them to school meetings with team IP planners, teacher, administrators to explain PCIT and what the family is doing in treatment	CLIN-SUPPORT
		Power Imbalance between the Therapist and Client	CLIN-CLIENT-POWER-IMBAL
	Adaptations/Adaptions	UC Davis PCIT	CLIN-ADAPT-UCD
		Extra Time in PDI Prep	CLIN-ADAPT-EX-PDI-PREP
		Extra Time in CDI Prep	CLIN-ADAPT-EX-CDI-PREP
		In-Person	CLIN-ADAPT-INP
		Telehealth	CLIN-ADAPT-TELE
		Clinician uses interpreter/translator (used to help code) Dr. McNeil Teach Powerpoints with Cultural Icon Touchpoint	CLIN-ADAPT-TRANSCLIN-ADAPT-MCNEIL-PP
		Make PCIT my own	CLIN-ADAPT-OWN
		General	CLIN-ADAPT-G
	Identity	Therapist Brings in Own Race/Identity as a Discuss/Model for Understanding PCIT within cultural context	CLIN-IDEN-MOD
		Therapist Uses Own Race/Identity to Discuss/Model Cultural Issues in therapy GENERALLY and broadly	CLIN-IDEN-DIS
Clinician Training		Training Culturally Insensitive/Race Not Included; OR the trainer demonstrated discomfort talking about cultural issues related to PCIT	CT-NO-CUL
		Trainers need be trained in cultural sensitivity	CT-TRAINER-CUL-Training
		Training was White Climate—Trainers and Trainees	CT-WHITE-CLIMATE
		Not expecting White Trainer to have information about Black Culture; Or just expected White trainer to provide information on PCIT	CT-WHT-TRAINER-No Expect
		PCIT Training—GEN	CT-PCIT-GEN
		Trainer was rigid about PCIT fidelity and strictly following treatment protocol	CT-PCIT-RIGID
		Power Differential (ex: Dual Role of Trainer in Academic Setting; Trying to Get Certified); especially when there is conflict in a opinion of how to manage a family; Some concerns that supervisor, especially if white, is advising things that could threaten the trainee’s relationship with the client	CT-POW-DIFF
		Trainee remarked that it was clear that they communicated and interacted differently from their trainers	CT-TRAINEE-CUL-DFRNT
		Trainer Talked about how to/was Receptive to be Flexible/Adaptive for Culture	CT-TRAINER-ADAPT
		Training was racist	CT-Racist
		Black Clinicians Fear Consequence for Speaking a Difference of Opinion or Challenging the Model in a Eurocentric Training Model; Or when they did speak, did not feel heard by the Trainer or that their opinions regarding culture were being respected.	CT-BLK-CLIN-SILENT
		Trainee raises issues of culture in the training.	CT-CLIN-CULTURE
		Trainer discussed all the ways PCIT was not suitable for addressing the concerns of BLack Families and stopped there, without discussing ways to make the tx tailored for Black families; and in some ways was excluding Black families and in this way was racists	CT-Libral Trainer—No flexible tailoring -Blk-FAM
		PCIT trainer was culturally sensitive and aware	CT-TRAINER-CUL
		PCIT trainer/training was good	CT-TRAINER-GOOD
		Openness/Flexibility of the Trainer; Need for flexibility	CT-TRAINER-OPEN
		Trainee needing to do their own process of figuring out PCIT would be a good fit for themselves as Clinicians—They hold a level of skepticism about whether PCIT will work—Cognitive Dissonance during Training	CT-COG-DIS/BUY-IN
		PCIT Training—Inadequate Other than Culture	CT-PCIT-INADEQ
		Time for Training is Too Time-Consuming and Conflicts with Regular Work Responsibility in not being able to Take that Time Off To Train	CT-TC
		Training is Expensive	CT-EXPENSE
		Received funding for PCIT training	CT-FUND
		Clinician suggested better agency support for the training, so it is not an additional burden to do the training. Or didn’t feel they had support w/getting a PCIT caseload.	CT-SUG-AGENCY-SUPPORT
		Training hard to complete due to getting sufficient caseload to graduate	CT-BARRIER-CASELOAD
		Clinician feared not being able to complete training	CT-FEAR-NOT COMPLETING
		PCIT Training Barriers due to COVID (“a lot of adjusting that I had to go through with the training process and so I kind of stumped a lot of my learning”.)	CT-BARRIERS-COVID
		PCIT Training was GENERALLY good	CT-PCIT-GOOD
		PCIT Training was average (70% range)	CT-PCIT-AVG
		PCIT Supervisor was busy, but could call on if you needed; not as much attention; thrown into doing things independently	CT-Supervisor-less hands on
		Following initial training had a good Supervisor trained in PCIT	CT-Good Supervisor
		Agency forced them to do the PCIT training when they didn’t want to	CT-CLIN-AGENCY-Cohersed
